# *Gardnerella vaginalis* alters cervicovaginal epithelial cell function through microbe-specific immune responses

**DOI:** 10.1186/s40168-022-01317-9

**Published:** 2022-08-04

**Authors:** Lauren Anton, Briana Ferguson, Elliot S. Friedman, Kristin D. Gerson, Amy G. Brown, Michal A. Elovitz

**Affiliations:** 1 Department of Obstetrics and Gynecology, Center for Research on Reproduction and Women’s Health, Perelman School of Medicine at the University of Pennsylvania, Philadelphia, PA 19104 USA; 2 Division of Gastroenterology and Hepatology, Perelman School of Medicine at the University of Pennsylvania, Philadelphia, PA 19104 USA; 3 Department of Microbiology, Perelman School of Medicine at the University of Pennsylvania, Philadelphia, PA 19104 USA

**Keywords:** Cervix, *Lactobacillus crispatus*, *Gardnerella vaginalis*, Epithelial barrier, Inflammation, TLR2, Preterm birth

## Abstract

**Background:**

The cervicovaginal (CV) microbiome is highly associated with vaginal health and disease in both pregnant and nonpregnant individuals. An overabundance of *Gardnerella vaginalis* (*G. vaginalis*) in the CV space is commonly associated with adverse reproductive outcomes including bacterial vaginosis (BV), sexually transmitted diseases, and preterm birth, while the presence of *Lactobacillus* spp. is often associated with reproductive health. While host-microbial interactions are hypothesized to contribute to CV health and disease, the mechanisms by which these interactions regulate CV epithelial function remain largely unknown.

**Results:**

Using an in vitro co-culture model, we assessed the effects of *Lactobacillus crispatus* (*L. crispatus*) and *G. vaginalis* on the CV epithelial barrier, the immune mediators that could be contributing to decreased barrier integrity and the immune signaling pathways regulating the immune response. *G. vaginalis*, but not *L. crispatus*, significantly increased epithelial cell death and decreased epithelial barrier integrity in an epithelial cell-specific manner. A *G. vaginalis*-mediated epithelial immune response including NF-κB activation and proinflammatory cytokine release was initiated partially through TLR2-dependent signaling pathways. Additionally, investigation of the cytokine immune profile in human CV fluid showed distinctive clustering of cytokines by *Gardnerella* spp. abundance and birth outcome.

**Conclusions:**

The results of this study show microbe-specific effects on CV epithelial function. Altered epithelial barrier function through cell death and immune-mediated mechanisms by *G. vaginalis*, but not *L. crispatus*, indicates that host epithelial cells respond to bacteria-associated signals, resulting in altered epithelial function and ultimately CV disease. Additionally, distinct immune signatures associated with *Gardnerella* spp. or birth outcome provide further evidence that host-microbial interactions may contribute significantly to the biological mechanisms regulating reproductive outcomes.

Video Abstract

**Supplementary Information:**

The online version contains supplementary material available at 10.1186/s40168-022-01317-9.

## Introduction

Host-microbe interactions play a significant role in the pathophysiology of human disease. Tissue-specific microbiomes are fundamental to host physiology and immunology. Interactions range from a mutual symbiosis (commensal) to disease-causing pathogenicity with many levels in between (contextual pathogen/pathobiont). Location-specific microbiomes have been shown to affect the host through multiple complex mechanisms including direct interactions with host cells, such as immune activation through bacterial cell well components or the release of microbial immune regulators including metabolites or antimicrobial proteins [1]. One of the most prominent and well-studied examples of host-microbe interactions occurs at mucosal surfaces overlaying epithelial barriers, which can be found in specific human niches including the gut [2, 3]. Cross talk between microbiota and host epithelial cells can promote or diminish the health of the epithelial barrier causing disease states such as inflammatory bowel disease (IBD) [4–6].

Similarly, the composition and diversity of the vaginal microbiome have been shown to be highly associated with cervicovaginal (CV) health and disease [7]. The vaginal microbiome has been well characterized in both nonpregnant and pregnant individuals using high-throughput 16S rRNA gene sequencing, and multiple community state types (CSTs) have been described [8–11]. Classification of CSTs has provided a systematic approach to studying the vaginal microbiome [8]. While there is variance in nomenclature, characterization of vaginal microbial communities is often defined by whether they are abundant or deficient in *Lactobacillus* spp. Consistent with this classification are CSTs that are dominated by *Lactobacillus* species including *L. crispatus* (CST-I), *L. gasseri* (CST-II), *L. iners* (CST-III), and *L. jensenii* (CST-V). CSTs dominated by *Lactobacillus* species, except for *L. iners* which is present in high abundance in women with BV, are often indicative of a healthy vaginal space and are characterized by the production of high levels of lactic acid (resulting in low vaginal pH) and bacteriocins produced by these bacteria [12, 13]. In contrast, CST-IV is deplete of *Lactobacillus* species and is instead composed of mostly strict and facultative anaerobic bacteria, such as *Gardnerella vaginalis*, *Mobiluncus*, *Fannyhessea vaginae* (previously known as *Atopobium vaginae*), *Prevotella* spp., and *Porphyromonas* spp. These microbes are typically associated with dysbiotic vaginal states such as bacterial vaginosis (BV) [14, 15], an increased risk of sexually transmitted diseases including human immunodeficiency virus (HIV) [16], and adverse reproductive outcomes [17, 18].

The vaginal microbiome has been associated with diverse aspects of female reproductive health including infertility, urinary tract infections, miscarriage, and adverse pregnancy outcomes, specifically spontaneous preterm birth (sPTB) [7, 19]. sPTB, or delivery prior to 37 weeks of gestation, is a complex multifactorial syndrome that remains poorly understood. While there are many factors that are associated with the development of sPTB, studies performed in the 1980s first identified a positive association between sPTB and the presence of BV [20–22]. Over the past 10 years, with the advancement of 16s rRNA gene sequencing technologies, studies have more robustly confirmed this association between sPTB and the CV microbiome providing evidence that a *Lactobacillus*-deficient vaginal microbiome could be a significant mechanistic contributor to the development of sPTB [9–11, 23, 24]. Data from a large, nested case-control study from our group showed significant associations between seven bacteria taxa, including *M. curtsii/mulieris*, *Fannyhessea*, and *Megasphaera* (all CST-IV), and increased risk of sPTB [10]. The risk of sPTB was positively associated with abundance of these specific taxa [8]. Even though a *Lactobacillus*-deficient CST-IV state may not be associated with a consistent clinical outcome, the presence of CST-IV confers a higher risk of adverse vaginal pathologies such as human papillomavirus (HPV), BV, HIV, and other sexually transmitted diseases [25–28]. Understanding the mechanisms by which *Lactobacillus*-deficient microbial communities impart an increased risk of adverse reproductive outcomes remains an obstacle to advancing reproductive health. Therefore, elucidating the host-microbe interactions within the CV space becomes necessary for targeted therapeutics to prevent and/or treat CV-based adverse reproductive outcomes such as BV, STIs, and sPTB.

sPTB is one of the most common adverse reproductive outcomes. While the essential mechanisms driving sPTB remain elusive, recent research ascribes a crucial role for the cervical epithelial barrier in sPTB. Breakdown of the cervical epithelial barrier is hypothesized to be an initiating step in the cervical remodeling process that occurs prior to delivery [29–32]. Multiple molecular factors have been shown to alter cervical epithelial barrier integrity including inflammatory mediators [33], cellular adhesion proteins [32–35], and miRNAs [32, 34, 36–38]. We propose that the CV microbiome is a common regulator of these molecular pathways. Previous studies published by our laboratory and others have shown that bacteria-free supernatants from *G. vaginalis* and *M. mulieris* cause breakdown of the ectocervical and endocervical epithelial barriers through cleavage of adhesion proteins, immune activation, and epigenetic regulation [31, 32]. Furthermore, mouse studies have shown that vaginal colonization with *G. vaginalis* results in sPTB [35]. While the results of these studies provide evidence that alterations in the CV microbiome can significantly alter epithelial cell function, they did not investigate the mechanistic drivers of host-microbe epithelial interactions.

In vivo, host-microbial interactions in the CV space are complex and likely driven by microbes, microbial by-products, and their communication with specific immune and epithelial cell types. As vaginal and cervical epithelial cells originated from different embryological origins [39–41], they likely have distinct cellular roles with complex cell-specific functions. Despite their unique origins, very few studies have investigated how host-microbe effects differ between cervical and vaginal cells [42, 43]. In vivo, both live bacteria and the molecules they secrete (e.g., proteins, metabolites, extracellular vesicles) may elicit relevant cellular effects, and it is unknown how these effects might be similar or divergent across CV epithelial cells. Therefore, revealing the interactions between bacteria and their by-products with different epithelial cell types within the CV space is necessary to advance our understanding of host-microbial interactions and their contributions to reproductive health and disease.

Therefore, the objectives of this study were (1) to determine the effects of live CV bacteria and their supernatants on the integrity of the cervical and vaginal epithelial barrier, (2) to elucidate the immune profile resulting from interactions between live bacteria or their supernatants and host CV epithelial cells, (3) to determine if toll-like receptor (TLR) signaling pathways are necessary for the activation of the epithelial cell immune responses from common CV microbes, and (4) to validate in vitro findings by assessing immune signatures in the CV space of pregnant individuals with a high abundance of *Gardnerella* spp.

## Materials and methods

### Cell culture

Ectocervical (Ect/E6E7, ATCC # CRL-2614) (Ecto), endocervical (End1/E6E7, ATCC CRL-2615) (Endo), and vaginal (VK2/E6E7, ATCC CRL-2616) (VK2) human epithelial cell lines (American Type Culture Collection, Manassas, VA) were cultured in keratinocyte-serum-free media (K-SFM) supplemented with 0.1 ng/mL epidermal growth factor and 50 ug/mL bovine pituitary extract (Gibco, Life Technologies), 100 U/mL penicillin, and 100 μg/mL of streptomycin at 37 °C in a 5% CO_2_ humidified incubator.

TLR2 (NF-kB-SEAP/KI-IL-8 Lucia) dual-reporter human embryonic kidney (HEK) 293 cells (Invivogen, San Diego, CA), a TLR2 reporter cell line, express the human TLR2 gene, an NF-kB/AP1-inducible SEAP (secreted embryonic alkaline phosphatase) reporter gene, and the Lucia luciferase reporter gene under the control of the endogenous IL-8 promoter. These cells also show no activity of TLR3, TLR5, and TNFR (tumor necrosis factor receptor). The HEK TLR2 cells were cultured according to the manufacture’s protocol. Briefly, HEK-TLR2 cells were grown in Dulbecco’s Modified Eagle’s Medium (DMEM, Mediatech, Corning, Glendale, AZ) containing 4.5 g/L glucose, 2 mM L-glutamine, 10% heat-inactivated fetal bovine serum (FBS, 30 min at 56 °C), 100 ug/mL Normocin (InvivoGen) and selective antibiotics — 100 ug/mL Hygromycin B Gold (InvivoGen), and 50 ug/mL Zeocin (InvivoGen). Cells were grown in 100 mm culture dishes at 37 °C in a 5% CO_2_ humidified incubator.

### Bacterial cultures and preparation of bacteria-free supernatants

Bacterial strains, *Lactobacillus crispatus* (ATCC 33197) or *Gardnerella vaginalis* (ATCC 14018), were obtained from the American Type Culture Collection (Manassas, VA). *G. vaginalis* was grown on human blood Tween bilayer agar plates (Fisher Scientific), and *L. crispatus* was grown on De Man, Rogosa, and Sharpe agar (Fisher Scientific); both strains were grown in New York City (NYC) III broth. Bacteria were grown at 37 °C in an anaerobic glove box (Coy Labs, Grass Lake, MI).

For each experiment, the following bacterial growth protocol was followed: *L. crispatus* and *G. vaginalis* glycerol stocks were streaked on agar plates and grown overnight. Individual colonies were used to inoculate starter cultures and grown overnight. Starter cultures were diluted to an optical density of 0.2 and then used to inoculate working cultures, which were grown for 20 h prior to use in experiments. Bacterial densities of the working cultures were estimated on the day of the experiment based on optical density readings at 600 nm using an Epoch 2 plate reader (Biotek, Winooski, VT), and the appropriate volume was centrifuged at 13,000 g for 3 min. The bacterial pellets were resuspended in K-SFM cell culture media without antibiotics and added to cells at 10^4^–10^6^ CFUs/well. Precise bacterial densities of the working culture were determined by CFU assays (plating serial dilutions of the working cultures). For all experiments, reported bacterial densities are +/− 0.5 log of the noted bacterial density (CFU/well).

To obtain supernatants, the working cultures were centrifuged at 13,000 g for 3 min, and the supernatant was filtered through a 0.22-μm filter (Fisher Scientific) to remove any remaining live bacteria. Bacteria-free supernatants are diluted to 10% v/v in K-SFM cell culture media without antibiotics. The final concentrations of bacteria-free supernatants per well (10^5^–10^7^ CFU/ml culture density) are reported based on the 10% v/v dilution of the bacterial culture density (CFU/ml) that the supernatants originated from.

### Epithelial cell/bacteria co-culture in vitro model

Ectocervical, endocervical, and vaginal cells were plated at 2.0 × 10^5^ cells/well in 24-well plates containing K-SFM without antibiotics. The next day, the cells were treated with either live *L. crispatus* or *G. vaginalis* (1 × 10^4^–1 × 10^6^ CFU/well) or 10% (v/v) bacteria-free supernatants (1 × 10^5^–1 × 10^7^ CFU/mL culture density) in K-SFM cell growth media for 24 h. For cells treated with bacteria-free supernatants from *L. crispatus*, K-SFM media was supplemented with 50 mM HEPES and sodium bicarbonate (3000 mg/L total concentration) to bring the pH of the media up to a physiological level (7.2) *as L. crispatus* bacteria produce high amounts of lactic acid during growth. Without pH adjustments, even at lower volume per volume percentages, the cells did not survive. In additional experiments, ectocervical, endocervical, and vaginal cells were pre-treated with a neutralizing IgA monoclonal antibody to human TLR2 (10ug/mL) (anti-hTLR2-IgA, InvivoGen, San Diego, CA) for 1 h prior to exposure to live bacteria or supernatants from *L. crispatus* or *G. vaginalis*. For all supernatant experiments, cells were also treated with NYCIII bacterial growth media alone as a negative control to determine any baseline effects of the growth media on the outcome of interest. At the end of each experiment, cell culture media was collected for cell death, ELISA or Luminex assays and/or the cells were collected in TRIzol (Invitrogen, Thermo Fisher Scientific) for RNA extraction.

### Differential interference contrast imaging

Ectocervical, endocervical, and vaginal epithelial cells were plated at 2.0 × 10^5^ cells/dish on 35 mm high glass bottom μ-dishes (ibidi, Martinsried, Germany) coated with 0.1% gelatin for 24 h. Live *L. crispatus* and *G. vaginalis* were added to the cells and incubated 4–6 h prior to imaging. Differential interference contrast (DIC) images of our epithelial cell/bacteria co-culture with *L. crispatus* and *G. vaginalis* were taken using the Zeiss Axio Observer 7 widefield microscope using the 100× objective (Zeiss 100×/1.4 NA oil Plan-Apochromat) with ZEN Blue software (version 2.5).

### HEK-hTLR2 treatments and NF-κB and IL-8 detection

HEK-hTLR2 cells were plated at 7.5 × 10^4^ cells/well in 96-well plates containing DMEM + 10% heat-inactivated FBS without antibiotics. The next day, the cells were treated with either live *L. crispatus* or *G. vaginalis* (10^4^–10^6^ CFU/well) or 10% (v/v) bacteria-free supernatants (10^7^–10^5^ CFU/mL culture density) in DMEM cell culture media for 24 h. In additional experiments, the cells were pre-treated with the TLR2 neutralizing antibody, anti-hTLR2-IgA (InvivoGen), for 1 h prior to exposure to live bacteria or supernatants. In these experiments, the TLR2 agonist FSL-1 (10 ng/mL, Sigma-Aldrich, St. Louis, MO) was used as a positive control for antibody efficacy. For detection of a nuclear factor kappa-B (NF-κB) response (SEAP reporter), cell culture supernatants were incubated with QUANTI-Blue solution (Invivogen) for 1 h, pictures were taken of the plate, and absorbance was read at 630 nm on a SpectraMax i3x plate reader (Molecular Devices). For detection of an IL-8 response (Lucia luciferase reporter), cell culture supernatants from the same experiment were combined with QUANTI-Luc solution (Invivogen), and luminescence was read immediately on a SpectraMax i3X plate reader. Additionally, cell culture supernatants were used in cell death assays as described below.

### Cell death assay

Ectocervical, endocervical, vaginal, and HEK-hTLR2 cells were grown as described above. The release of lactate dehydrogenase (LDH) from ectocervical, endocervical, and vaginal cells (*n* = 3–9 independent experiments per cell type) was measured using the CytoTox 96 nonradioactive cytotoxicity assay (Promega, Madison, WI). This assay allows for the quantitative measurement of LDH that is released upon cell lysis using a coupled enzymatic assay that results in the conversion of a tetrazolium salt into a red formazan product. The amount of color formed is proportional to the amount of LDH released as the cells lyse. Color formation was read on a colorimetric plate reader at 490 nm, and absorbance values were recorded.

### Cell permeability experiments

Ectocervical, endocervical, and vaginal cell permeability was determined using an In Vitro Vascular Permeability Assay (Millipore, Bedford, MA). Briefly, ectocervical, endocervical, and vaginal cells were plated at 1.0 × 10^6^ cells/mL into 24-well hanging cell culture inserts which contain 1 μm pores with a transparent polyethylene terephthalate (PET) membrane pre-coated with collagen. The cells were treated with either live *L. crispatus* or *G. vaginalis* (10^6^–10^4^ CFU/well) or 10% (v/v) bacteria-free supernatants from *L. crispatus* (*n* = 6) and *G. vaginalis* (*n* = 9) for 24 h. In additional experiments, ectocervical, endocervical, and vaginal cells were pre-treated with the anti-hTLR2 antibody prior to exposure to live bacteria or supernatants from *L. crispatus* or *G. vaginalis* (as described above) for 24 h (*n* = 9). For all supernatant experiments, cells were also treated with NYCIII bacterial growth media alone as a negative control to determine any baseline effects of the growth media on cell permeability. After 24 h of treatment, the media was removed, and phenol red free K-SFM media (ScienCell Laboratories, Carlsbad, CA) containing FITC-dextran was added to the top of the insert. The movement of FITC-dextran from the top insert to the bottom was measured after 2 h by a fluorescent plate reader at 485 nm and 535 nm, excitation and emission, respectively.

### ELISA

Ectocervical, endocervical, and vaginal cells were cultured in 24-well plates and treated with either live bacteria or bacteria-free supernatants as stated above. IL-8 was measured in cell culture media after 24 h of treatment. The expression of IL-8 was measured by a ligand-specific commercially available ELISA kit that utilizes a quantitative sandwich enzyme immunoassay technique using regents from R&D systems (Minneapolis, MN).

### Luminex assay

Ectocervical, endocervical, and vaginal cells were cultured and treated with live bacteria or bacteria-free supernatants as stated above. A 41-plex cytokine/chemokine (HCYTMAG-60K-PX41) and a TGFB 3-plex (TGFBMAG-64K-03) human magnetic bead Luminex panel (EMD Millipore, Billerica, MA) were run on (1) ectocervical, endocervical, and vaginal cell culture media after 24 h of treatment with *L. crispatus* (*n* = 3) or *G. vaginalis* (*n* = 3) live bacteria or bacteria-free supernatants and (2) cell culture media from ectocervical, endocervical, and vaginal cells pre-treated with an anti-TLR2 blocking antibody followed by *L. crispatus* (*n* = 3) or *G. vaginalis* (*n* = 3) live bacteria and 3) human cervical vaginal fluid (CVF) collected from individuals with or without a sPTB with low or high CV *Gardnerella* spp. abundance. Human CVF was collected using Dacron swabs (Starplex, Thermo Fisher). Materials on the swabs were eluted in sterile PBS with a protease inhibitor cocktail (Complete Mini) for 5 min to release the soluble proteins. All samples were run in duplicate, per the manufacturer’s protocol on the FLEXMAP 3D Luminex platform (Luminex, Austin, TX). Absolute quantification in pg/mL was obtained using a standard curve generated by a five-parameter logistic (5PL) curve fit using xPONENT 4.2 software (Luminex). Fold change values were calculated between treatment groups and the non-treated control (NTC) for live bacteria or between the treatment groups and the NYC media control for bacteria-free supernatants. For those cells pre-treated with the anti-TLR2 blocking antibody, fold change was calculated between the non-treated control and bacteria treatment alone or the bacteria plus the anti-TLR2 antibody. For fold change calculations, if the cytokines levels in the control group were undetectable, then a minimal detectable level was assigned equal to 10% of the mean cytokine level. For human samples, data is expressed as average pg/mL. Heatmaps were created using R.

### mRNA isolation from epithelial cells

Following treatment with live *L. crispatus* and *G. vaginalis*, ectocervical, endocervical, and vaginal cells were collected in TRIzol (Invitrogen, Thermo Fisher Scientific) and underwent phenol-chloroform extraction. The resulting aqueous phase was further column purified with the miRNeasy kit (Qiagen, Hilden, Germany) according to the manufacturer’s protocol for total RNA isolation. RNA concentration was determined via a NanoDrop 2000 Spectrophotometer (NanoDrop™ Rockland, DE) prior to the generation of cDNA.

### cDNA generation and qPCR

cDNA was generated from 1 μg of isolated RNA from ectocervical, endocervical, and vaginal cells using the High-Capacity cDNA Reverse Transcription Kit (Applied Biosystems, Thermo Fisher Scientific). qPCR was performed on the 7900HT Real-Time PCR System (Applied Biosystems) using the TaqMan Universal PCR Master Mix (Applied Biosystems) according to the manufacturers’ protocols. The standard curve method was used for relative expression quantification using the RQ manager software v2.4 (Applied Biosystems). The relative abundance of the target of interest was divided by the relative abundance of 18S in each sample to generate a standardized abundance for the target transcript of interest. All mRNA primers were purchased from Applied Biosystems: TLR2, MYD88, NOD1, NOD2, and 18S.

### Human samples

To investigate if the cytokine signature in pregnant individuals with high and low *Gardnerella* spp. abundance was different, we utilized CVF samples from a prospective cohort study of 2000 individuals with singleton pregnancies. Details of this study are available in the primary report [10]. All participants provided written informed consent, and the study was approved by the Institutional Review Board at the University of Pennsylvania (IRB no. 818914) and the University of Maryland School of Medicine (HP-00045398). Cervicovaginal specimens were self-collected by the participant or collected by a research coordinator between 16 and 20 weeks. A set of cervicovaginal swabs was obtained including an ESwab (COPAN) stored in 1 mL of Amies transport medium and a Dacron swab stored without buffer. All samples were immediately frozen at −80 °C until processing. All delivery outcomes were recorded. Cases of PTB were closely monitored to determine if they were true spontaneous deliveries, and not secondary to other maternal/fetal disease, as previously described [10]. To assess the CV microbial communities, 16s rRNA gene sequencing were performed as described previously [10].

For this study, a nested case-control selection of participants was performed (Table 1). Inclusion criteria were individuals identified as being in CST IV at 16–20 weeks by microbial sequencing (*n* = 131). From this cohort of individuals with CST IV, four groups were identified by birth outcome and *Gardnerella* spp. abundance. As 16s rRNA gene sequencing cannot distinguish between different species of *Gardnerella* and to account for the fact that multiple *Gardnerella* species have been recently identified [44, 45], we refer to this as *Gardnerella* spp. moving forward in the results and discussion. The four groups were (1) term with low *Gardnerella* spp. (*Gardnerella* abundance lower than 0.2), (2) sPTB with low *Gardnerella* spp., (3) term with high *Gardnerella* spp. (*Gardnerella* abundance of 0.2 or greater), or (4) sPTB with high Gardnerella spp. *Gardnerella* spp. abundance cutoff of 0.2 was determined based on the calculated median from the whole cohort with the lower confidence limit of the median defined as a “low value.”

**Table 1:** Patient demographics

Maternal characteristics	All (***n*** = 131)	sPTB low ***Gardnerella***	Term low ***Gardnerella***	sPTB high Gardnerella	Term high ***Gardnerella***	***P***-value
Race						0.138
White	12 (9.2)	1 (4.4)	3 (6.5)	4 (20.0)	4 (9.5)	
Black	118 (90.1)	22 (95.7)	43 (93.5)	15 (75.0)	38 (90.5)	
Other	1 (0.8)	0 (0.0)	0 (0.0)	1 (5.0)	0 (0.0)	
Age (mean, SD)	26.6 (5.8)	27.7 (5.7)	26.6 (5.7)	26.3 (6.1)	26.2 (5.8)	0.76*
Gestational age at delivery (mean, SD)	36.0 (6.1)	27.8 (6.5)	39.2 (0.9)	30.9 (6.3)	39.4 (0.8)	< 0.001*

Presented as *n* (col %); chi-square *p*-value unless otherwise indicated

*sPTB* spontaneous preterm birth, *GV Gardnerella vaginalis*

*Kruskal-Wallis test

### Statistical analysis

Statistical analyses were performed for all experiments with the GraphPad Prism software (Version 9.0, San Diego, CA). For data that were normally distributed, one-way analysis of variance (ANOVA) was performed. If statistical significance was reached (*p* < 0.05), then pairwise comparison with a Tukey post hoc test was performed for multiple comparisons. If data were not normally distributed, then the nonparametric Kruskal-Wallis test was used, and pairwise comparison was done using Dunn’s multiple comparison test. Analysis of Luminex cytokine (pg/ml) data (shown as fold change in the heatmaps) was performed using a one-way ANOVA followed by different post hoc tests comparing treatment groups to (1) a non-treated control (NTC) was done using a Dunnett’s multiple comparison test or (2) between select pairs of treatment groups was done using Sidak’s multiple comparisons test. Human CVF Luminex data were analyzed by two-tailed *t*-test with term delivery compared to sPTB in both the low and high *Gardnerella* spp. abundance groups. If the variances between groups were significantly different, then a *t*-test with Welch’s correction was used.

## Results

### *G. vaginalis* co-localizes with cervicovaginal epithelial cells resulting in increased cell death in a host-microbial co-culture model

**Fig. 1:**
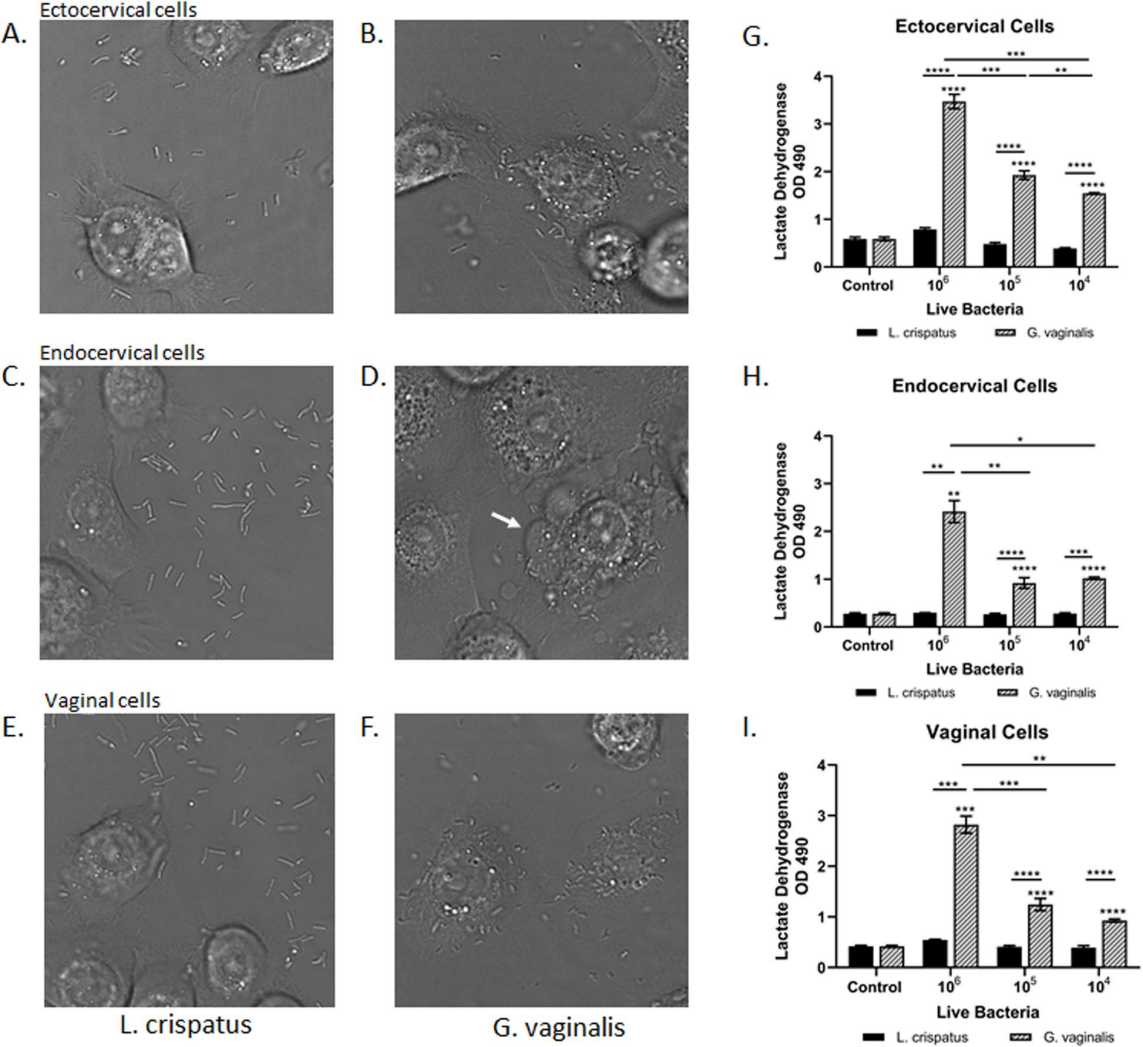
Co-localization of cervicovaginal epithelial cells with live *G. vaginalis* but not *L. crispatus* results in increased cell death. An in vitro live bacteria and host cervicovaginal co-culture model were created to study host microbial interactions in the CV space. Representative images of ectocervical (**A**, **B**), endocervical (**C**, **D**), and vaginal (**E**, **F**) epithelial cells interacting with *L. crispatus* (**A**, **C**, **E**) or *G. vaginalis* (**B**, **D**, **F**) are shown. Exposure of ectocervical (**G**), endocervical (**H**), and vaginal (**I**) cells to *G. vaginalis* but not *L. crispatus* results in dose-dependent cell death after 24 h. Values are mean ± SEM. Asterisks over the individual bars represent comparisons with control; asterisks over solid lines represent comparisons between treatment groups. *p < 0.05, **p < 0.01, ***p < 0.001, ****p < 0.0001

In creating our in vitro host-microbial co-culture model, characterization of bacterial interactions with host epithelial cells was necessary before moving forward with subsequent experiments. Differential interference contrast (DIC) images of *L. crispatus* and *G. vaginalis* co-cultured with ectocervical (Fig. 1A, B), endocervical (Fig. 1C, D), and vaginal (Fig. 1E, F) cells showed limited co-localization between the epithelial cells and *L. crispatus* (Fig. 1A, C, E). However, there was significant co-localization/interaction between the CV epithelial cells and *G. vaginalis* (Fig. 1B, D, F). Co-culture of *G. vaginalis* with all three cell types resulted in observed cell blebbing indicative of cellular stress and death. Cytotoxicity assays confirmed the observed cell death in all three cell lines (Fig. 1G, H, I). Lactate dehydrogenase (LDH) was increased in a dose-dependent manner (10^4^–10^6^ CFU/well) after co-culture with *G. vaginalis* (but not *L. crispatus*) in ectocervical (*p* < 0.0001), endocervical (*p* < 0.0001), and vaginal (*p* < 0.0001) cells. LDH was unchanged in all three cell lines after exposure to bacteria-free supernatants from *L. crispatus* or *G. vaginalis* (Supplemental Fig. 1).

### *L. crispatus* and *G. vaginalis* have divergent effects on cervical and vaginal epithelial barrier permeability

**Fig. 2:**
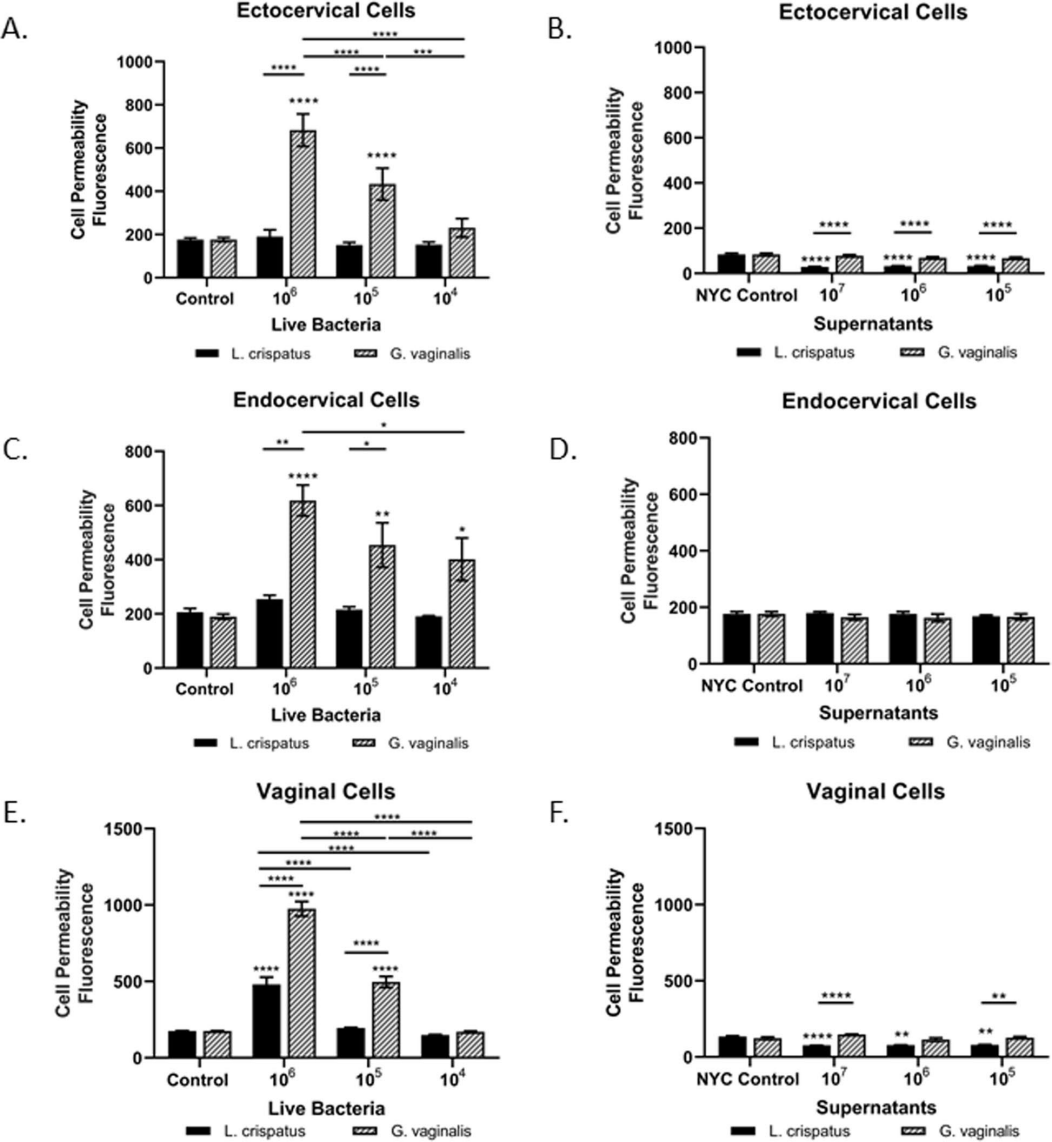
Live *G. vaginalis* increases epithelial barrier permeability. Cell permeability was measured in ectocervical, endocervical, and vaginal epithelial cells after 24-h exposure to live bacteria (**A**, **C**, **E**) or bacteria-free supernatants (**B**, **D**, **F**) of *L. crispatus* or *G. vaginalis*. Bacterial growth media alone acted as a negative control for the bacteria-free supernatants tested. Cell permeability is expressed as fluorescence OD measurements from a fluorescent plate reader and is indicative of the movement of FITC-dextran from the top to the bottom insert of a transwell chamber system. Values are mean ± SEM. Asterisks over the individual bars represent comparisons with control; asterisks over solid lines represent comparisons between treatment groups. *p < 0.05, **p < 0.01, ***p < 0.001, ****p < 0.0001

To determine if the presence of live *L. crispatus* or *G. vaginalis* or their supernatants alone is essential for functional alteration of the cervical and/or vaginal epithelial cell barriers, cell permeability assays were performed in ectocervical, endocervical, and vaginal epithelial cells. Live *G. vaginalis* significantly increased cell permeability in a dose-dependent manner in all three cell lines (ecto: *p* < 0.0001, endo: *p* < 0.001, vaginal: *p* < 0.0001), with no significant effects seen with live *L. crispatus* at the same doses (Fig. 2A, C, E). CV cells co-cultured with live *G. vaginalis* had significantly higher cell permeability when compared to those co-cultured with *L. crispatus* at both the 10^6^ (ecto: *p* < 0.0001, endo: *p* = 0.0072, vaginal: *p* < 0.0001) and 10^5^ (ecto: *p* < 0.0001, endo: *p* = 0.0233, vaginal: *p* < 0.0001) doses of bacteria but not at 10^4^ (CFU/well). Exposure of ectocervical, endocervical, and vaginal cells to bacteria-free supernatants from *G. vaginalis* had no effect on cell permeability at any of the doses tested, while *L. crispatus* supernatants significantly decreased cell permeability, when compared to the NYC control, similarly at all three doses tested (ecto: *p* < 0.0001, endo: NS, vaginal: *p* < 0.0001) (Fig. 2B, D, F). There was no significant effect of the bacterial growth media alone (NYC control) on cell permeability of ectocervical, endocervical, or vaginal cells.

### *G. vaginalis* and *L. crispatus* bacteria and their bacterial-free supernatants induce distinct immune profiles in CV epithelial cells

**Fig. 3:**
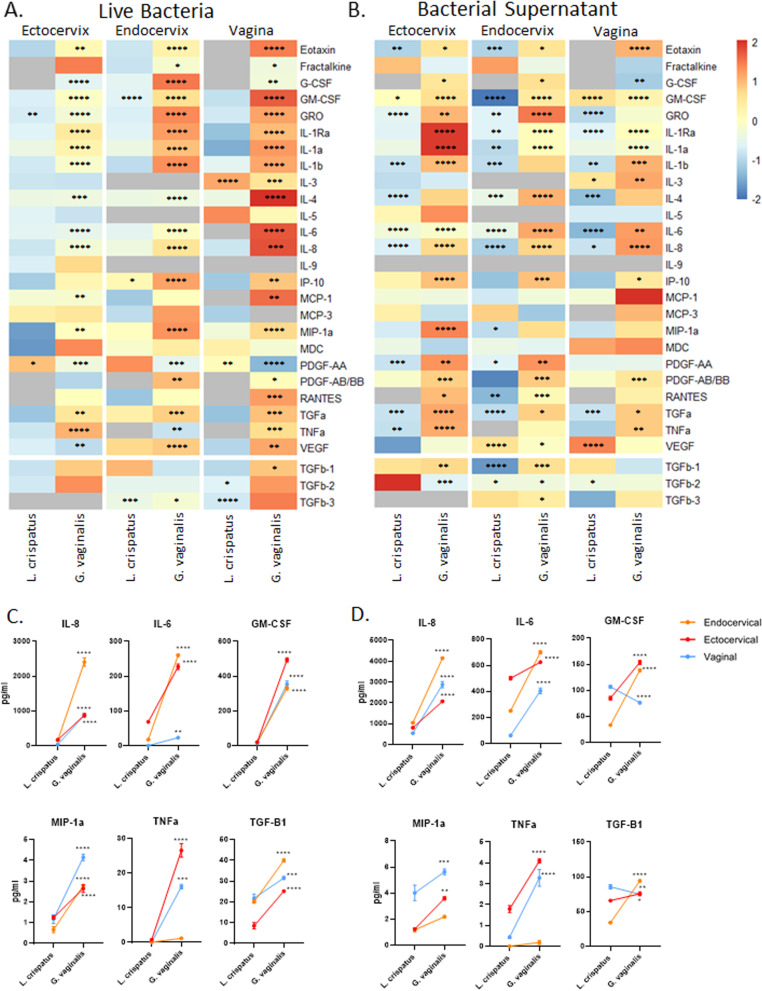
Live *G. vaginalis* activates the host-epithelial immune response, while bacteria-free supernatants alter immune activation in a nonbacterial-specific manner. Immune cytokines/chemokines released from ectocervical, endocervical, and vaginal cells after exposure to live *L. crispatus* and *G. vaginalis* (**A**) or their bacteria-free supernatants (**B**) for 24 h were measured by Luminex. Heat map depicts fold change (vs NTC for live bacteria or vs NYC for bacteria-free supernatants) by color and *p*-value by asterisks. *p*-value is based on pg/ml values. Representative graphs of cytokines (pg/mL) showing significant differences between *L. crispatus* and *G. vaginalis* exposure for both live bacteria (**C**) and bacteria-free supernatants (**D**) show epithelial cell-specific responses. Values are mean ± SEM. *p < 0.05, **p < 0.01, ***p < 0.001, ****p < 0.0001

We have previously shown that *G. vaginalis* bacteria-free supernatants can increase the release of a varied group of inflammatory mediators from ectocervical cells [32]; however, the contribution of live bacteria (including bacterial cell wall and its associated proteins) to activating a host-epithelial cell immune response was not assessed. Therefore, we sought to examine the effects of both live bacteria and bacteria-free supernatants on the activation of the immune response from all three CV epithelial cell lines. Of the 44 cytokines/chemokines included in the discovery-based Luminex arrays (41-plex immune and 3-plex TGFB), 28 were detectable in at least one of the three cell lines after exposure to live bacteria (Fig. 3A), while 27 were detectable after exposure to bacteria-free supernatants (Fig. 3B). A full list of all cytokines/chemokines included in the Luminex assays and their average values (pg/mL) and standard deviations in each cell type can be found in Supplemental Table 1A (live bacteria) and B (bacteria-free supernatants). In cases where samples had very low cytokine expression, when only one of the three biological replicates was detectable (and the others were undetectable, defined as out of range low), this treatment group was denoted as not- detected (ND) in Supplemental Tables 1A and B. However, if at least two of the biological replicates gave a detectable reading, then the average of the detectable readings (pg/ml) was reported in Supplemental Tables 1A and B. Most of the detectable cytokines were significantly increased after exposure to live *G. vaginalis* versus exposure to *L. crispatus* (when each are compared to NTC) (Supplemental Table 2). PDGF-AA was the only cytokine decreased after live *G. vaginalis* exposure in all three cell lines. Very few analytes were altered by live *L. crispatus* with two, three, and four cytokines changed in ectocervical, endocervical, and vaginal cells, respectively (Supplemental Table 2A). In cells exposed to bacteria-free supernatants, there were significant numbers of cytokines altered in both the *L. crispatus* and *G. vaginalis* groups. In cells exposed to bacteria-free supernatants from *L. crispatus*, the majority of altered cytokines were decreased, while in cells exposed to *G. vaginalis*, the majority of altered cytokines were increased (Supplemental Table 2B). Overall, similar cytokines/chemokines were detected after co-culture with either live bacteria or bacteria-free supernatants; however, the levels of those cytokines varied widely between the two exposures with 13 cytokines being higher after exposure to live bacteria when compared to supernatants. Nine were lower after live bacteria exposure compared to supernatants, and five were similar. A representative subset of cytokines (Fig. 3C, D) demonstrates the differential cell-specific responses of ectocervical, endocervical, and vaginal cells to either live bacteria (Fig. 3C) or bacteria-free supernatants (Fig. 3D). Graphs for all detectable cytokines demonstrating cell-specific differences in cytokine levels after exposure to either *L. crispatus* or *G. vaginalis* (live and supernatants) can be found in Supplemental Fig. 2.

### *L. crispatus* and *G. vaginalis* bind to TLR2-activating NF-κB

**Fig. 4:**
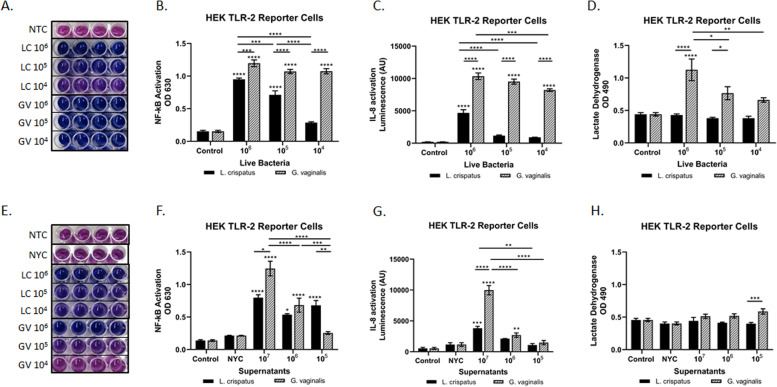
*L. crispatus* and *G. vaginalis* activate NF-κB signaling through TLR2; however, only *G. vaginalis* results in increased IL-8 levels. The HEK TLR2 reporter cell line was used to determine if either live or bacteria-free supernatants from *L. crispatus* and *G. vaginalis* activated TLR2-mediated cell signaling. Representative images (**A**, **E**) and the corresponding quantification (**B**, **F**) of the QUANTI-Blue NF-κB detection assay, IL-8 activation (SEAP quantification) (**C**, **G**), and cytotoxicity (**D**, **H**) were all altered after exposure to live bacteria and bacteria-free supernatants from *L. crispatus* and *G. vaginalis*. For **A** and **E**, darker blue/purple indicates higher NF-kB. Values are mean ± SEM. Asterisks over the individual bars represent comparisons with control; asterisks over solid lines represent comparisons between treatment groups. *p < 0.05, **p < 0.01, ***p < 0.001, ****p < 0.0001

To identify the intracellular immune pathways being activated by *G. vaginalis*, we utilized HEK TLR2 reporter cells. Live *G. vaginalis* and *L. crispatus* induced NF-κB activation in a dose-dependent manner with *G. vaginalis*-exposed cells sustaining a higher level (than *L. crispatus*) of NF-κB across all three bacterial doses tested (Fig. 4A, B). IL-8 activation was significantly elevated in a dose-dependent manner to live *G. vaginalis* but not *L. crispatus* (except at the 10^6^ dose) (Fig. 4C). A similar response was seen after exposure to bacteria-free supernatants from *G. vaginalis* and *L. crispatus* (Fig. 4E–G)*.* HEK TLR2 cell death was similar to that seen in the CV epithelial cells with a dose-dependent increase in LDH in live *G. vaginalis* co-cultures and no significant difference found after exposure to live *L. crispatus* nor any of the bacteria-free supernatants (Fig. 4D, H).

### *G. vaginalis* mediates increased cytokine release partially through TLR2-activated signaling pathways

**Fig. 5:**
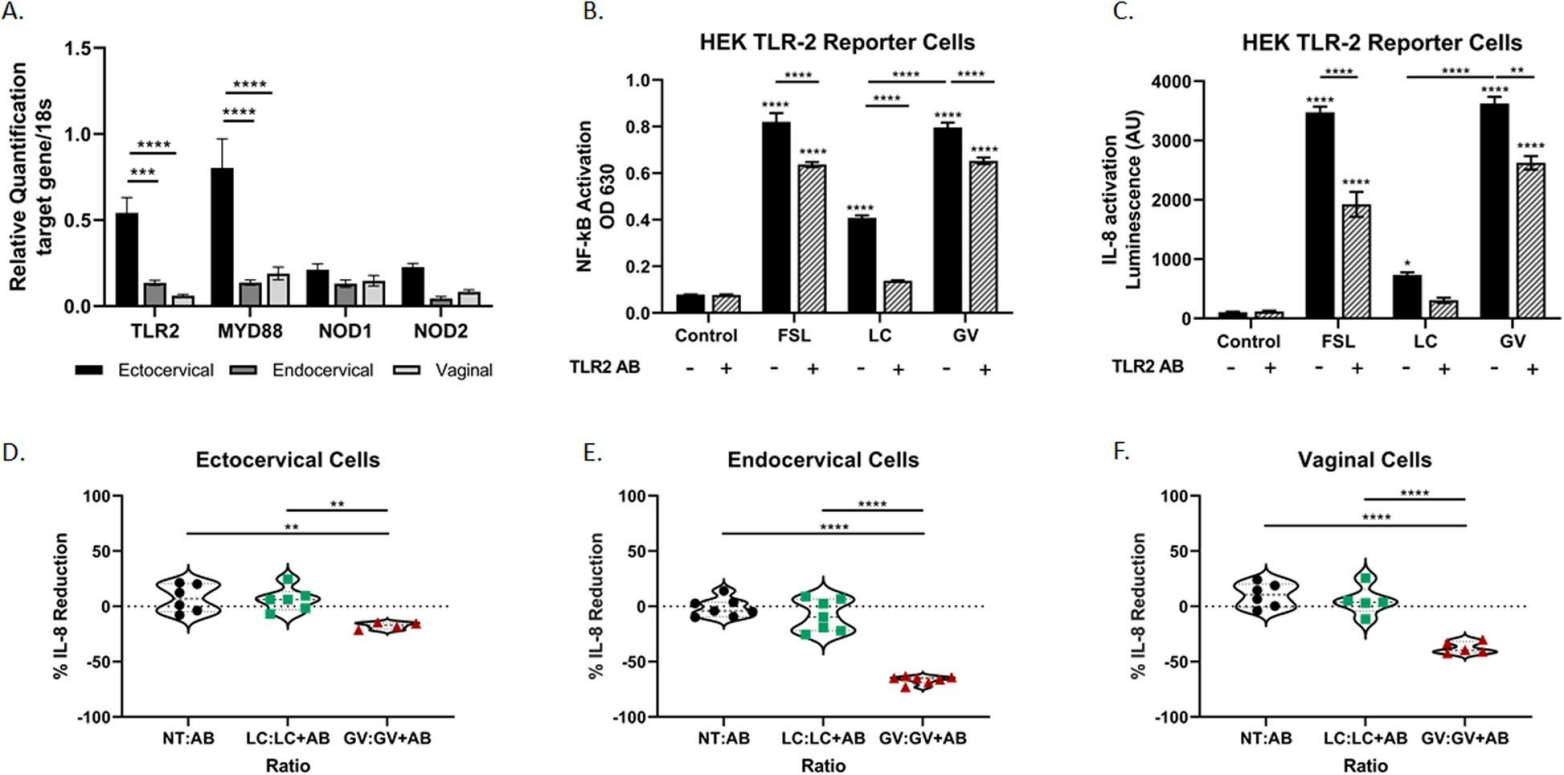
Blocking the TLR2 receptor significantly reduces *L. crispatus* and *G. vaginalis*-induced NF-κB and IL-8 activation. TLR2, MYD88, NOD1, and NOD2 are expressed in cervicovaginal epithelial cells, but expression varies by epithelial cell type (**A**). Blocking the TLR2 receptor in the HEK TLR2 reporter cells significantly reduced *L. crispatus* and *G. vaginalis*-induced NF-κB activation (**B**) and only *G. vaginalis*-induced IL-8 (**C**). The TLR2 agonist FSL was included as a positive control. Blocking the TLR2 receptor in cervicovaginal epithelial cells resulted in a reduction in *G. vaginalis*-induced IL-8, but no effect was seen in *L. crispatus* treated cells (**D**, **E**, **F**). Data is expressed as a percent reduction of the ratio of treatment alone to treatment plus anti-TLR antibody (**A**, **B**). Values are mean ± SEM. Asterisks over the individual bars represent comparisons to control; asterisks over solid lines represent comparisons between treatment groups. *p < 0.05, **p < 0.01, ***p < 0.001, ****p < 0.0001

While this study has shown that NF-κB and a multitude of cytokines are activated/increased in response to live *G. vaginalis*, at least partially, through activation of the TLR2 receptor, we have not investigated if the TLR2 receptor is a necessary activation signal in the CV immune response. TLR2, MYD88, NOD1, and NOD2 are all expressed in ectocervical, endocervical, and vaginal epithelial cell lines (Fig. 5A). The expression of TLR2 (*p* = 0.0007 versus endo, *p* < 0.0001 versus vaginal) and MYD88 (*p* < 0.0001 versus endo, *p* < 0.0001 versus vaginal) is significantly higher in ectocervical cells when compared to endocervical and vaginal cell lines. Both NOD1 and NOD2 were equally expressed across cell lines. To determine if TLR2 activation is essential for the epithelial immune response and consequent cytokine increase, we blocked the TLR2 receptor and examined cytokine output after exposure to *L. crispatus* and *G. vaginalis* in both HEK TLR2 cells and all three CV cell lines. In HEK TLR2 cells, FSL (a potent TLR2 agonist, *p* < 0.0001), *L. crispatus* (*p* < 0.0001), and *G. vaginalis* (*p* < 0.0001)-mediated increases in NF-κB were significantly reduced with TLR2 blockade (Fig. 5B). Similarly, pretreatment with the anti-TLR2 antibody also reduced IL-8 activation after exposure to FSL (*p* < 0.0001) and *G. vaginalis* (*p* = 0.0007) but not *L. crispatus* (Fig. 5C). Preliminary investigation into the *G. vaginalis*-mediated increase in cytokine expression in CV cell lines showed that blocking TLR2 also significantly reduced IL-8 expression in a cell-type-specific manner: 17% reduction in ectocervical cells (*p* = 0.0067, Fig. 5D), 66% reduction in endocervical cells (*p* < 0.0001, Fig. 5E), and 37% reduction in vaginal cells (*p* < 0.0001, Fig. 5F).

**Fig. 6:**
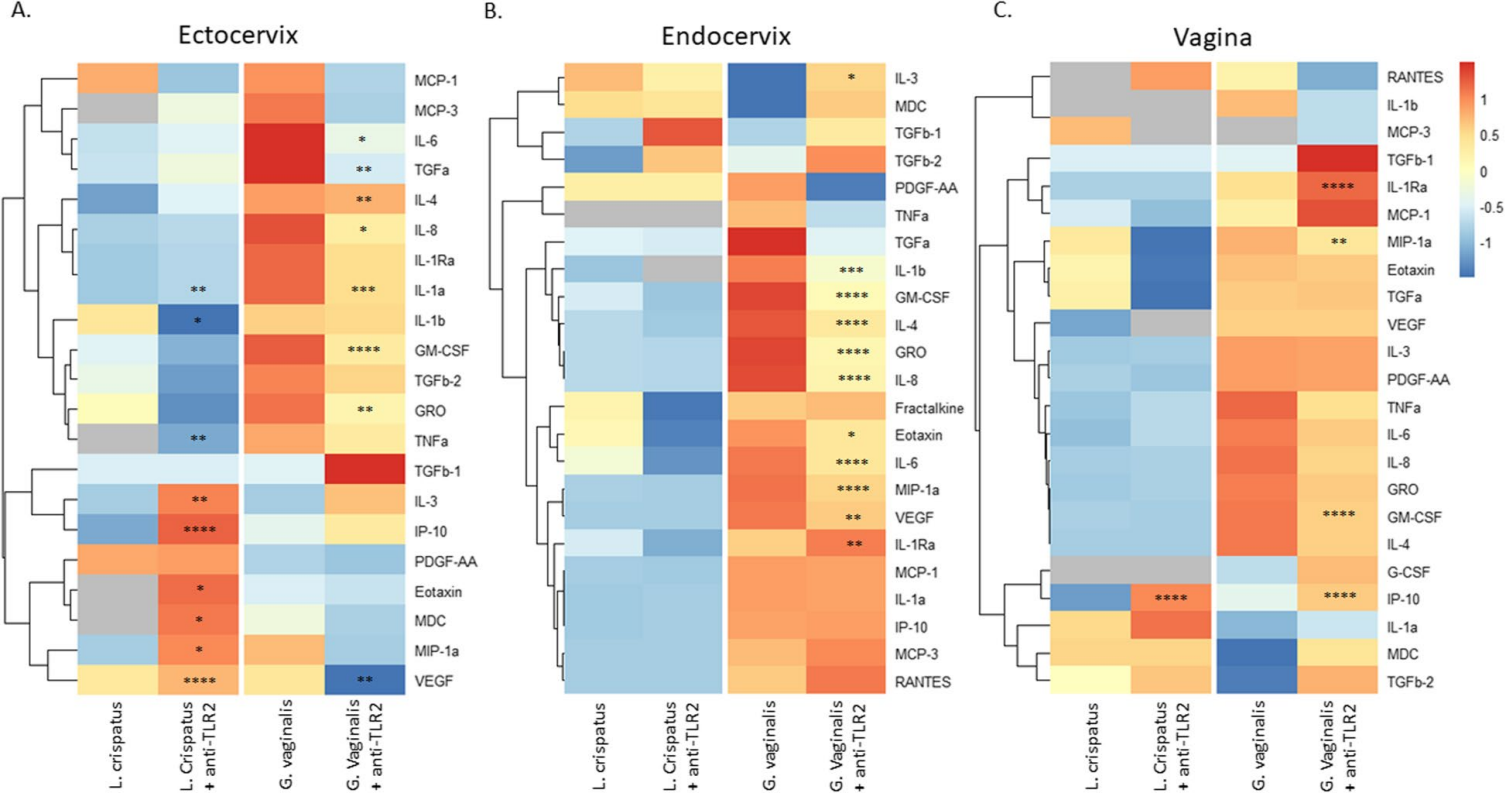
Immune profile of cervicovaginal epithelial cells identifies cytokines mediated by activation of TLR2. Blocking the TLR2 receptor alters the immune cytokines/chemokines released from ectocervical (**A**), endocervical (**B**), and vaginal cells (**C**) after exposure to live *L. crispatus* and *G. vaginalis*. Heat map depicts fold change by color and *p*-value by asterisks. Fold change was calculated between the non-treated (NTC) control and live bacteria alone or live bacteria plus anti-TLR2 antibody. *p*-value is based on pg/ml values. *p < 0.05, **p < 0.01, ***p < 0.001, ****p < 0.0001

To further investigate the role of TLR2 activation in *G. vaginalis*-mediated cytokine production in all the CV cell lines, we assessed the immune response after TLR2 blockade using the same Luminex panels as described above (Fig. 6A, B, C). As we observed previously, there was a varied immune response between the three cell lines with ectocervical cells exhibiting the greatest overall number of cytokines altered after TLR2 blockade in both *L. crispatus* and *G. vaginalis* exposed cells followed by endocervical and then vaginal cells (Supplemental Table 3). TLR2 blockade inhibited the *G. vaginalis*-induced increase in cytokine expression across all cell lines, except for IL-1RA which was increased. In contrast, TLR2 blockade only mitigated the *L. crispatus*-induced cytokine expression in ectocervical but not endocervical or vaginal cells (Fig. 6). Interestingly, TLR2 blockade also increased seven *L. crispatus*-induced cytokines in ectocervical cells and one cytokine in vaginal cells.

### Activation of TLR2 is not essential for the *G. vaginalis*-mediated breakdown of the cervical and vaginal epithelial barrier

**Fig. 7:**
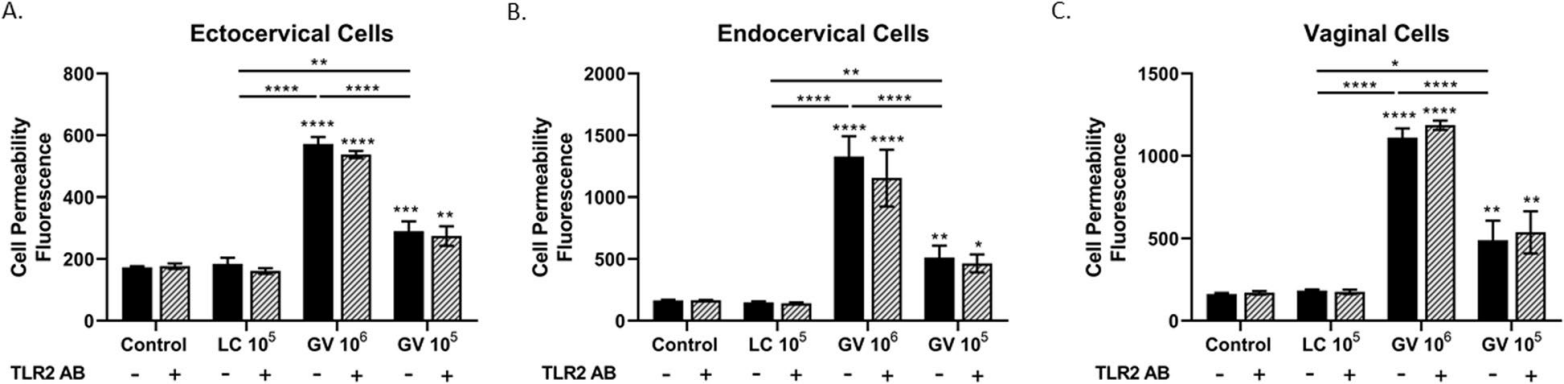
Blocking the TLR2 receptor does not mitigate *G. vaginalis*-induced increases in cell permeability. Cell permeability was measured in ectocervical (**A**), endocervical (**B**), and vaginal (**C**) epithelial cells after pretreatment with the anti-TLR2 antibody and subsequent exposure to live bacteria. *G. vaginalis*-induced cell permeability was unchanged after blocking the TLR2 receptor. Values are mean ± SEM

Since we have evidence that live *G. vaginalis* induces a significant immune response through the activation of TLR2, we wanted to determine if the TLR2 receptor is an essential mechanism in the observed loss of integrity of the cervical and vaginal epithelial barrier. Blocking the TLR2 receptor in ectocervical (Fig. 7A), endocervical (Fig. 7B), and vaginal (Fig. 7C) epithelial cells did not mitigate the ability of live *G. vaginalis* to breakdown the epithelial barrier at either of the two doses of *G. vaginalis* tested.

### Pregnant individuals with a high CV *Gardnerella* spp. abundance who deliver preterm have a distinct CV immune signature from those with a term birth

**Fig. 8:**
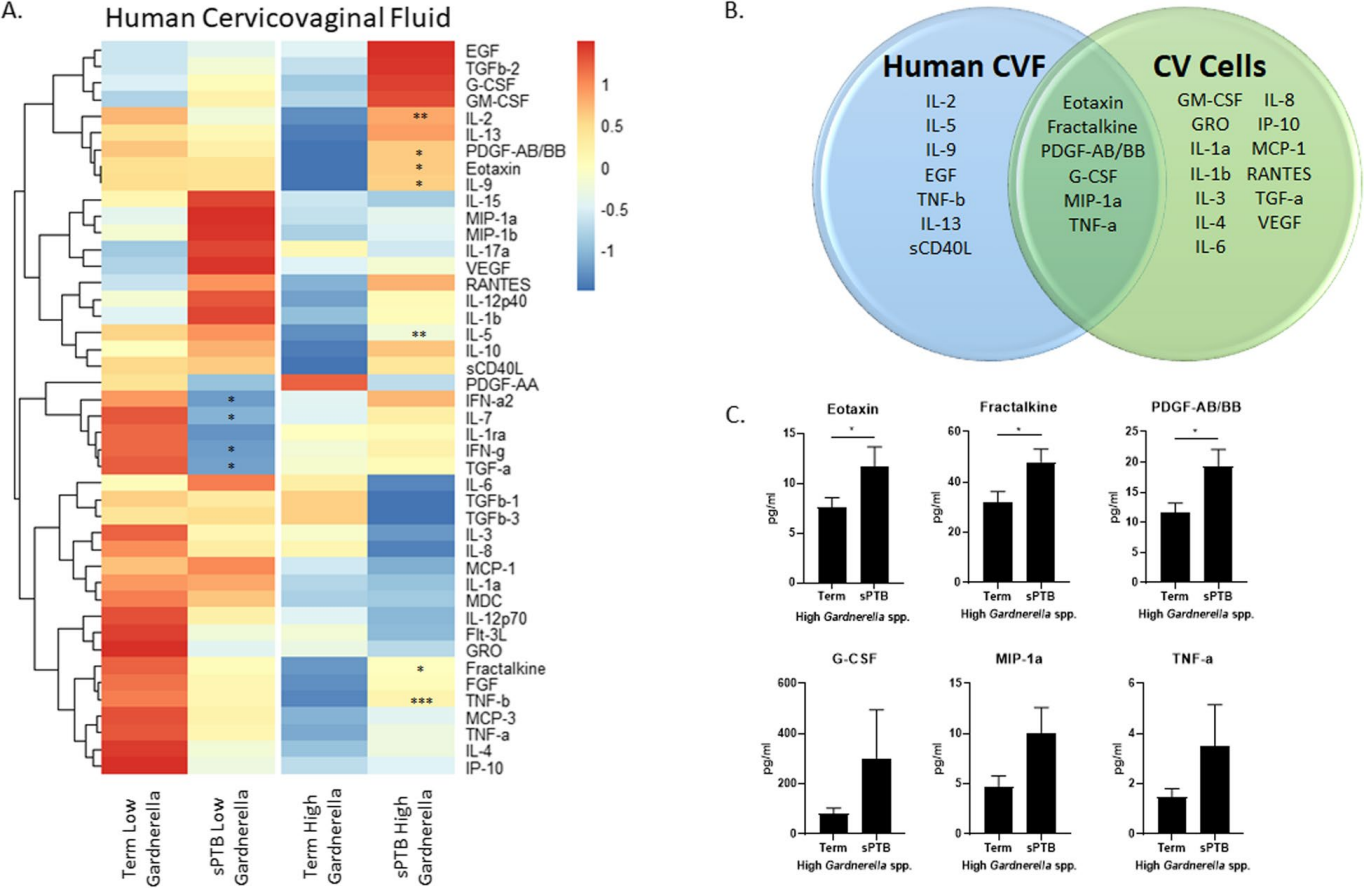
Immune profile from human cervicovaginal fluid is altered by abundance of *Gardnerella* spp. and delivery outcome. Cervicovaginal swabs collected from CST IV pregnant individuals at 16–20 weeks of gestation with either term or sPTB deliveries were used for Luminex assays to determine immune profiles of cytokines/chemokines. Heat map shows immune profiles vary by both *Gardnerella* spp. abundance as well as delivery outcome (**A**). Ven diagram shows overlapping cytokines (*p* < 0.10) induced in CVF from individuals with high *Gardnerella* spp. abundance and sPTB versus cervicovaginal epithelial cells exposed to live *G. vaginalis* in culture (**B**). Graphs of overlapping cytokines (**C**). Heat map (**A**) values are based on pg/mL and expressed on a relative scale. Values are mean ± SEM. *p < 0.05 and a trend for significance = *p* < 0.10

To investigate if *Gardnerella* spp. induces a distinct immune signature in those who ultimately have a sPTB versus term birth, we performed Luminex on CVF from 131 pregnant individuals categorized into CST-IV by 16S rRNA gene sequencing [10]. Four groups comprised of individuals with high or low *Gardnerella* spp. CVF abundance and term or sPTB delivery outcomes were analyzed. Secondarily, the CVF immune profile of individuals with high *Gardnerella* spp. abundance and a sPTB was compared against the immune profile our in vitro co-culture model (CV epithelial cells exposed to live *G. vaginalis*). All 44 cytokines studied were detectable in human CVF. Of those 44, seven were significantly (*p* < 0.05) increased in individuals with high CVF *Gardnerella* spp. abundance who underwent a preterm delivery compared to those that delivered at term (Fig. 8A). Six additional cytokines (EGF, G-CSF, IL-13, MIP-1a, TNF-a, sCD40L) were non-significantly increased (*p* < 0.10) in those who had a sPTB and high *Gardnerella* spp. abundance. Four cytokines were significantly decreased (*p* < 0.05) in CVF from individuals with a low *Gardnerella* spp. abundance who had a sPTB compared to those with a term birth (Fig. 8A). Of the 20 cytokines that were significantly increased in the *G. vaginalis* epithelial cell co-culture model (Fig. 3A), six cytokines overlapped between human CVF from individuals with high *Gardnerella* spp. and a sPTB and CV epithelial cells exposed to live *G. vaginalis* (Fig. 8B and C).

## Discussion

This study demonstrates the complexity of host-microbial interactions within the cervicovaginal space and provides evidence that CV commensal bacteria, *L. crispatus* and *G. vaginalis*, have differential effects on epithelial function. We have shown that *G. vaginalis*, a vaginal microbe commonly associated with adverse reproductive health, results in both functional and immune-based host responses as evidenced by increased CV epithelial cell death, epithelial barrier breakdown, and immune activation partially through TLR2/NF-κB-induced expression of chemokines/cytokines. However, *L. crispatus*, a microbe associated with optimal vaginal health, does not induce changes in epithelial cell death nor epithelial barrier function. We reveal that vaginal and cervical epithelial cells elicit varying immune responses to similar microbe challenges and, thus, emphasize the need to understand the complexity of the CV space for optimizing reproductive health. Importantly, these studies demonstrate that activation of TLR2 is a shared pathway across epithelial cell types in response to *G. vaginalis* exposure. Results from our human study confirm our in vitro findings that the host-microbe-mediated CV immune response is associated with the abundance of select microbes and notably that an increased cytokine response is associated with birth outcome when *Gardnerella* spp. abundance is high.

In both pregnant and non-pregnant individuals, the primary function of the cervical and vaginal epithelial cells is to create a barrier against harmful microbes. To accomplish this, multiple diverse mechanisms are used including the creation of a physical barrier made up of the epithelial cells themselves, the expression of adhesion and tight junction proteins responsible for holding the cells tightly together, and the production of a mucous layer that protects the epithelial cells by entrapping or killing pathogens. Pathogenic microbes have historically developed ways to circumvent this barrier, as is the case with *G. vaginalis*, which expresses a number of factors that enhance its virulence potential including adherence to CV epithelial cells, biofilm forming capabilities, mucous degradation (sialidase) [46], and host cell cytotoxicity [47]. *G. vaginalis* has been shown to produce vaginolysin [48, 49], a cholesterol-dependent cytolysin that acts as a cytotoxic pore-forming toxin and, consequently, kills host epithelial cells. Similar to previous studies investigating the cytotoxic effects of *G. vaginalis* [50], we observed CV cell death in our in vitro host-microbial co-culture model providing evidence that *G. vaginalis* functional outcomes were conserved in this model. Interestingly, the cytotoxic effect of *G. vaginalis* was similar across all three epithelial cell types with a slightly more potent effect in ectocervical versus endocervical and vaginal cells. *L. crispatus*, as expected, had no effect on CV epithelial cell death despite being exposed to a large dose (10^6^ CFU/well) of bacteria. *L. crispatus* seems to interact with CV epithelial cells to a lesser extent than was observed with *G. vaginalis*, as evidenced by the DIC images (Fig. 1A–F). However, this decreased adherence did not prevent it from activating immune/molecular pathways, where exposure to live or bacteria-free supernatants of *L. crispatus* can activate NF-κB, increase IL-8, and other cytokines. In fact, direct interaction of the whole bacteria with epithelial cells is not needed to induce a host response as recent studies have identified *Lactobacillus*-secreted factors such as metabolites [51], hydrogen peroxide [52], and extracellular vesicles [53, 54] as having direct effects on cellular function.

The integrity of the CV barrier is essential not only to vaginal health (vaginal epithelial barrier) but also to a successful pregnancy (cervical epithelial barrier). The breakdown of the cervical epithelial barrier is thought to be a critical step in the cervical remodeling and dilation process that occurs prior to the onset of labor [55]. Consequently, early disruption of the epithelial barrier is hypothesized to contribute to preterm delivery through premature cervical remodeling or microbe-associated inflammatory mechanisms. A previous study from our laboratory supports this hypothesis as it demonstrated that intravaginal *G. vaginalis* colonization in a pregnant mouse model results in preterm birth with an activated immune response, breakdown of the epithelial barrier, induced cervical remodeling, and altered cervical biomechanics [35]. Consistent with our previous findings in an animal model, we show that live *G. vaginalis* decreases epithelial barrier integrity in vitro in all three CV cell types. We have previously demonstrated that *G. vaginalis* bacteria-free supernatants (factors secreted by bacteria) are also able to breakdown the cervical epithelial barrier [32]. In contrast to this prior report, in our current study, we found that bacteria-free supernatants did not have the same effect on epithelial cell permeability. One notable difference is the exposure time used in this study (24 h) versus our previous study (48 h). While exposure time may be a cause of the difference in findings with bacteria-free supernatants, it is also worth considering the more potent effects of live bacteria on epithelial cell function (predominantly increased cell death) as this likely occurs in vivo. While there was a significant difference in both ectocervical and vaginal (but not endocervical) cell permeability between *G. vaginalis* and *L. crispatus* bacteria-free supernatant exposed cells, the effect of *G. vaginalis* supernatants was diminished compared to live *G. vaginalis*. These results do provide evidence that live bacteria (and their cell wall) may be a more effective or significant contributor to epithelial barrier function than bacteria-secreted factors alone. However, a key finding of these experiments is that microbes and their supernatants elicit different biological effects on CV epithelial cells. In vivo, both the live bacteria and their secreted factors likely contribute to the CV host response. For example, vaginolysin, which is most likely present in both live bacteria and bacteria-free supernatant treatment groups, will contribute significantly to the breakdown in the epithelial cell barrier due to increased cell death. Therefore, to fully understand the biological pathways that govern vaginal/reproductive health, it will be essential to reveal the harmful and beneficial effects of both live bacteria and their supernatants. As such, further investigation into the composition (e.g., proteins, metabolites) of bacteria-free supernatants would be warranted to fully understand their role in regulating CV epithelial function.

A breakdown in the CV barrier likely has multiple overlapping causes. Therefore, while epithelial cell death definitively plays a significant role in decreased barrier integrity, there are other mechanisms that have been shown to contribute to a breakdown in the cervical and vaginal epithelial barriers including inflammation [33]. As part of the epithelial barrier, one of the main functions of epithelial cells is to initiate an innate immune response to fight invading pathogens. Several previous studies from our laboratory, and others, have shown that *G. vaginalis* (live and supernatants) induces a robust but widely varied and complex inflammatory response in CV epithelial cells [32, 56–58]. A previous study investigated cytokine production by CV epithelial cells after exposure to commensal microbes and BV-associated bacteria (BVAB), specifically live *Lactobacillus johnsonii*, *Lactobacillus vaginalis*, *G. vaginalis*, and *Fannyhessea* (*Atopobium) vaginae*. They found that BV-associated bacteria, including *G. vaginalis*, induced a varied innate immune response across the three CV epithelial cell types with IL-6, IL-8, G-CSF, IP-10, MIP-1β, RANTES, and GRO-α increased after BVAB exposure [57]. Similarly, in this current study, the presence of live bacteria revealed a microbe-specific immune profile. *G. vaginalis* exposure resulted in the activation of an immune response, with similar cytokines being increased, across all three CV epithelial cells lines; however, the level of cytokine production across the three cell types was often diverse and varied by each cytokine analyzed. At a macroscopic level, vaginal epithelial cells seemed to have a more robust innate response to live *G. vaginalis* when compared to the cervical epithelial cells. This finding suggests that vaginal epithelial cells may act as a first line of defense against pathogens entering the vaginal canal and may help to protect the cervix from infection, especially during pregnancy. The immune response evoked by *G. vaginalis* bacteria-free supernatants was also robust in activating cytokines, and like our previous published study [32], *L. crispatus* also significantly altered cytokine production albeit mostly in a downward direction indicative of its commensal, and perhaps protective, functions. While *L. crispatus* supernatant exposure predominately resulted in a decrease in cytokine levels, in our previous study where we first identified alterations in cytokine production by bacterial supernatants, we found that *L. crispatus* increased several cytokines. This difference in findings may be due to the longer exposure time (48 h) in our previous study. How these findings correlate/mimic the human physiological exposure of epithelial cells to microbes within the CV space would require further studies as the effects of the length of microbial exposure on CV epithelial immune function have not been extensively studied. Overall, these results indicate that CV epithelial cells can recognize multiple different bacterial components, either from bacterial cell wall or secreted factors, and mount an essential immune response. We hypothesize that this type of redundancy makes it more difficult for harmful bacteria to evade host immune recognition.

Epithelial cell recognition of bacterial components has been shown to activate the innate immune response through toll-like receptor (TLR) signaling. Of the TLRs, TLR2 is known to be activated by pathogen-associated molecular patterns (PAMPs) including lipoproteins which are ubiquitous to all bacteria and highly expressed in the outer membrane of Gram-positive bacteria. Once activated, TLR2 initiates intracellular signaling pathways which induce nuclear translocation of NF-κB to modulate gene transcription and consequent inflammatory cytokine production and immune cell infiltration [59, 60]. Previous studies have shown that CV lavage samples from clinical cases of BV upregulate cytokines through TLR2-mediated mechanisms [61, 62]. There is a paucity of data regarding the role of TLR signaling in the setting of CV host-microbe interactions. A previous study investigating CV epithelial cell immune responses showed the presence of TLR2 in both primary and immortalized ectocervical, endocervical, and vaginal cells, and that FSL-1 activation of the TLR2 receptor caused varying upregulation of IL-1B, IL-6, IL-8, and MCP-1 across the three cell lines [58]. The results of that study largely agree with our cytokine profile of increased IL-1B, IL-6, IL-8, and MCP-1 (among others) after exposure to *G. vaginalis* (live and supernatants). To expand on these findings, using a TLR2 reporter cell line, we found that exposure to live and bacteria-free supernatants of *G. vaginalis* significantly induced NF-κB and IL-8. Interestingly, *L. crispatus* also activated NF-κB, but that activation did not result in increased IL-8, except at the highest bacterial inoculum. These results indicate that high doses of bacteria, independent of bacteria species, activate a TLR2-mediated immune response. The non-specific activation of TLR2 by assumed healthy and non-healthy microbes may be a regulatory mechanism by the CV epithelium to prevent bacterial overgrowth. Alternatively, it is possible that a secondary signal or that a threshold of NF-κB activation may be needed to activate cytokine production in response to *L. crispatus* or other CV microbes. The ability of *G. vaginalis* to cause host immune activation at a dose where *L. crispatus* does not suggest that host cells are able to recognize bacteria-specific PAMPs and mount an immune response to more pathogenic bacteria. Additionally, like our cell death and permeability results, live bacteria exposure, compared to bacterial supernatants, results in a more robust IL-8 response, again indicating that whole bacteria have more substantial effects on these cellular functions than bacterial-secreted factors alone.

It is well known that immune system activation is a redundant process with the ability of many different signals/receptors to be activated resulting in the same outcome of upregulated cytokines which also have overlapping functions (cytokine pleiotropy) [63]. Therefore, identifying which factors within the immune response are essential for altering CV epithelial cell function becomes necessary to target these pathways for future therapeutic or preventative strategies against conditions associated with non-optimal microbial communities in the CV space such as BV, STI acquisition, or sPTB. Since we have shown in this study, along with others [58], that CV epithelial cells express TLR2 and the adaptor protein, MYD88, needed for NF-κB activation [60, 64, 65], we investigated if TLR2 activation is an essential mechanism for bacteria-induced host immune activation. After blocking the TLR2 receptor, the reduction in NF-κB activation seen with both live *L. crispatus* and *G. vaginalis* exposure also resulted in a mitigated IL-8 response. The complete mitigation of *L. crispatus*-induced NF-κB and IL-8, but not *G. vaginalis*, indicates that TLR2-mediated immune activation may not be the only immune pathway activated by *G. vaginalis*. Interestingly, in CV epithelial cells, blocking TLR2 resulted in varying levels of IL-8 reduction depending on the cell type. The biggest reduction in IL-8 (66% after blocking TLR2) was seen in endocervical cells. This result agrees with the results of our Luminex assay, where the greatest number of *G. vaginalis*-induced cytokines was reduced after blocking TLR2 in endocervical cells perhaps indicating that TLR2 signaling is a bigger contributor to the immune response in endocervical cells than the other cell types. Of note, all three CV cell types express NOD1 and NOD2 which recognize intracellular PAMPs that can enter the cell through phagocytosis or membrane pores (such as those created by vaginolysin). NODs are part of the NLRP3 inflammasome which can activate NF-κB signaling independently of membrane-bound TLRs. *G. vaginalis* has been shown to activate the NLRP3 inflammasome in macrophages and monocytes [66, 67]. Therefore, it is possible that NOD signaling may be a significant regulator of *G. vaginalis*-mediated inflammation in CV cells, especially in ectocervical and vaginal cells where TLR2 is likely not the only mediator of host-microbial mediated inflammation.

While the TLR2 blockade inhibited *G.* *vaginalis*-mediated epithelial immune activation, it did not prevent *G. vaginalis*-induced changes in epithelial cell permeability. Our data suggest that while TLR2 receptor blockade prevents some immune response, it is insufficient to limit the diverse downstream molecular effects from exposure to *G*. *vaginalis*. Consistent with the redundancy of the host immune response, it is plausible that multiple pathways need to be blocked to significantly mitigate the *G. vaginalis*-mediated breakdown of the CV epithelial barrier. In addition to inflammation, other biological mechanisms are known to regulate the epithelial barrier including cell death and tight junctions; therefore, it is quite likely that a combination of interconnected factors are necessary for a functional change to the barrier. Interestingly, epithelial cells can detect the presence of bacterial pore-forming cytotoxins, such as vaginolysin, at very low concentrations (sub-cytolytic) which acts as an early warning signal to activate an immune response [68]. Vaginolysin can activate the p38 MAP kinase pathway and induce the production of IL-8 independent of TLR2 activation [48]. Therefore, future research targeting a combination of biological mechanisms would be warranted to fully assess the essential mechanisms regulating CV epithelial barrier function.

We acknowledge that an in vitro model of host-microbial interactions has its limitations mostly due to the nature of a single cell type acting alone without the effects of surrounding cells including stromal cells and immune effector cells (neutrophils, T cells, NK cells, macrophages). Therefore, we chose to compare our in vitro cytokine results with those seen in a well-characterized human cohort of CVF samples collected from pregnant individuals characterized as having CST-IV, with high or low *Gardnerella* spp. abundance, who ultimately had a term delivery or sPTB [10]. Although human data inherently has wide variability, the immune signature revealed a distinctive clustering of cytokines within each group analyzed. Interestingly, there seems to be a larger number of elevated cytokines in CVF from pregnant individuals with low compared to high *Gardnerella* spp. abundance early in pregnancy. Since all individuals in this study have a CST-IV microbial state, these results would indicate that the interaction of select CST-IV microbes (and not *Gardnerella* spp. alone), within the community and/or the metabolic output of the community, plays an important role in regulating the CV immune response. Historically, studies investigating *G. vaginalis* and the immune response mostly in vaginal samples from individuals with BV have found significant increases in inflammatory cytokines including IL-8, IL-6, IL-1a, and IL-1b [57, 69–71]. One of these previous studies investigated the effect of BV-associated bacteria on the innate immune response in the CVF of BV-positive, nonpregnant women and found increased cytokines including IL-8 and IL-1b with trends for increased IL-6 and G-CSF which was similar to the cytokine profile seen in CV epithelial cells exposed to *Fannyhessea vaginae* [57]. In recent years, there has been some debate as to whether *G. vaginalis* initiates a significant inflammatory response, especially in cases of asymptomatic BV [72]. It has been suggested that *G. vaginalis* in combination with other CST-IV or other BV-associated bacteria (*Fannyhessea vaginae* and *Sneathia* spp.) are needed to cause significant clinical or inflammatory symptoms [72, 73]. Part of this discrepancy could also be due to the identification of more/less virulent strains (biotypes/ecotypes) of *G. vaginalis* which are associated with BV status and symptoms [74, 75]. In this cohort, unlike previous studies [57], we did not categorize patients by BV status, but by *Gardnerella* spp. abundance and delivery outcome instead; therefore, it is difficult to determine if the *Gardnerella* spp. subtypes present in these individuals fall into the more virulent category. However, it is interesting to note that when comparing individuals with high *Gardnerella* spp. abundance who had a term versus sPTB, there is a cluster of elevated cytokines (EGF, G-CSF, GM-CSF, IL-2, PDGF, Eotaxin, and IL-9) that may give some insight into identifying those individuals who are at higher risk for sPTB. A more in-depth analysis of this data including correlations with patient metadata, other bacterial species abundance, and immune regulators is planned in future studies.

In comparing the immune signature from our human dataset to that of our in vitro host-microbial co-culture model, we found both similarities and differences in the baseline presence and elevation of the cytokines. Not surprisingly, there were more detectable cytokines in human cervicovaginal samples when compared to our in vitro cultures (44 vs 28 cytokines). Additionally, there was some overlap in elevated cytokines between the high *Gardnerella* spp. abundance with sPTB group and our CV cells exposed to live *G. vaginalis* (six cytokines) indicating that our in vitro co-culture partially mimics the activated immune response seen in human samples. Of note, IL-8 was not increased in the high *Gardnerella* spp. abundance group in our human cohort. While this was surprising given the strong correlation observed with IL-8 and *G. vaginalis* exposure in our in vitro cultures, we acknowledge that the inflammatory profiles between a single-cell culture and a true physiological response will be different. While IL-8 is often measured as a common marker of an activated immune response, there is not consistent agreement regarding which cytokines are essential microbial drivers of the host-microbial immune cross talk that occurs within the CV space. However, the primary outcome of an activated *G. vaginalis*-mediated inflammatory response is similar in both sample subsets Given this result, the in vitro co-culture model remains a useful tool in studying the mechanisms regulating host-microbial interactions with the knowledge that findings using this model should be verified in human samples as well.

### Limitations

The results of this study provide evidence that the presence of both live *G. vaginalis* and its supernatant can induce specific epithelial immune responses across the cervicovaginal epithelial barrier. We acknowledge that the use of a single epithelial cell co-culture model cannot fully recapitulate in vivo conditions present in the pregnant human CV space. However, a single-cell model does allow for in-depth investigation into the fundamental mechanistic pathways regulating the epithelial immune response in the presence of select microbial species. Future studies using more advanced in vitro systems (cervicovaginal organoids, primary cell lines, organ-on-a-chip) would be warranted to more closely mimic a human CV phenotype. Given the recent studies identifying multiple *Gardnerella* spp. and those showing that certain strains may be more/less virulent, our findings with the *G. vaginalis* (ATCC) strain may be similar or divergent to other *G. vaginalis* strains. Studies utilizing clinical isolates of *G. vaginalis* with different virulence factors (cytotoxicity, biofilm formation, immune activation, etc.) will help to elucidate how different strains may initiate similar or differing immune responses. Additionally, we use two microbes to represent CV health and disease. While this is a reductionist approach, these results provide a platform on which to study other CV microbes and their supernatants to gain a better understanding of how they modulate the CV epithelium. With increased understanding of how each bacterial species alters CV epithelial function, multispecies microbial communities, which more aptly mimic the human condition, can be utilized to comprehensively reveal how competing or commensal communities may alter epithelial immune responses and drive reproductive health.

## Conclusions

Overall, the results of this study have provided critical insight into the host-microbial interactions between CV epithelial cells and the microbiota that inhabit the CV space. This study has shown that *G. vaginalis* alters cervical and vaginal epithelial cell function by decreasing epithelial barrier integrity, increasing cell death, and activating an inflammatory response that is partially regulated by TLR2/NF-κB-based signaling pathways. This study shows distinct epithelial cell-specific immune responses to microbe-specific signals that reveal complex interactions within the CV space. Understanding differences in the epithelial immune response in the cervix versus vagina will also provide insights into cervical or vaginal specific immune-based therapies/treatments for known *G. vaginalis*-associated states such as BV, sexually transmitted diseases (e.g., HIV), or adverse pregnancy outcomes including preterm birth. Additionally, differential immune profiling of CST-IV dominant pregnant individuals with altered levels of *Gardnerella* spp. abundance and delivery outcomes provides an immune signature for risk stratification of individuals who may ultimately have a sPTB. The results from this study begin to ascribe a biological mechanism as to how host epithelial cells and CV microbes interact to regulate cellular function in both commensal and dysbiotic states.

## Supplementary Information

**Additional file 1: Supplemental Figure 1.** Lactate Dehydrogenase levels after cervicovaginal epithelial cell exposure to 10% (v/v) bacteria-free supernatants from *Lactobacillus crispatus* or *Gardnerella vaginalis* (1x10^5^-1x10^7^ CFU/mL culture density) for 24 h. **Supplemental Figure 2.** Cytokine levels (pg/ml) after 24hr exposure to live bacteria or bacteria-free supernatants in ectocervical (red), endocervical (yellow) or vaginal (blue) epithelial cells showing epithelial cell specific responses. **Supplemental Table 1A.** Cytokine values (pg/ml) in cervicovaginal cells co-cultured with live *L. crispatus* (LC), *G. vaginalis* (GV) or non-treated control (NTC). **Supplemental Table 1B.** Cytokine values (pg/ml) in cervicovaginal cells exposed to *L. crispatus* (LC), *G. vaginalis* (GV) bacteria-free supernatant or NYC media alone (NYC). **Supplemental Table 2A.** Number of altered cytokines after exposure to live bacteria. **Supplemental Table 2B.** Number of altered cytokines after exposure to bacteria-free supernatants. **Supplemental Table 3.** Number of cytokines altered after blocking TLR2.

## Data Availability

All data generated or analyzed during this study are included in this published article and its supplementary information files.

## Abbreviations

CV: Cervicovaginal
CST: Community state type
BV: Bacterial vaginosis
HIV: Human immunodeficiency virus
sPTB: Spontaneous preterm birth
TLR: Toll-like receptor
Ecto: Ectocervical epithelial cells
Endo: Endocervical epithelial cells
VK2: Vaginal epithelial cells
K-SFM: Keratinocyte serum-free media
HEK: Human embryonic kidney cells
NYC: New York City III bacterial growth media
LDH: Lactate dehydrogenase
