# Supplementary material for: Gardnerella vaginalis alters cervicovaginal epithelial cell function through microbe-specific immune responses
Source: Microbiome. 2022 Aug 4;10:119. doi: 10.1186/s40168-022-01317-9 (PMC9351251; doi:10.1186/s40168-022-01317-9)
Supplement: Supplementary file 2 — Additional file 1: Supplemental Figure 1. Lactate Dehydrogenase levels after cervicovaginal epithelial cell exposure to 10% (v/v) bacteria-free supernatants from Lactobacillus crispatus or Gardnerella vaginalis (1x10^5^-1x10^7^ CFU/mL culture density) for 24 h. Supplemental Figure 2. Cytokine levels (pg/ml) after 24hr exposure to live bacteria or bacteria-free supernatants in ectocervical (red), endocervical (yellow) or vaginal (blue) epithelial cells showing epithelial cell specific responses. Supplemental Table 1A. Cytokine values (pg/ml) in cervicovaginal cells co-cultured with live L. crispatus (LC), G. vaginalis (GV) or non-treated control (NTC). Supplemental Table 1B. Cytokine values (pg/ml) in cervicovaginal cells exposed to L. crispatus (LC), G. vaginalis (GV) bacteria-free supernatant or NYC media alone (NYC). Supplemental Table 2A. Number of altered cytokines after exposure to live bacteria. Supplemental Table 2B. Number of altered cytokines after exposure to bacteria-free supernatants. Supplemental Table 3. Number of cytokines altered after blocking TLR2. [file 40168_2022_1317_MOESM1_ESM.docx]

**Supplemental Figure 1:** Lactate Dehydrogenase levels after cervicovaginal epithelial cell exposure to 10% (v/v) bacteria-free supernatants from *Lactobacillus crispatus* or *Gardnerella vaginalis* (1x10^5^-1x10^7^ CFU/mL culture density) for 24 hours. Values are mean ± SEM.

**Live Bacteria Bacterial Supernatant**

**Live Bacteria Bacterial Supernatant**

**Supplemental Figure 2:** Cytokine levels (pg/ml) after 24hr exposure to live bacteria or bacteria-free supernatants in ectocervical (red), endocervical (yellow) or vaginal (blue) epithelial cells showing epithelial cell specific responses. Statistical analysis compares *L. crispatus* vs *G. vaginalis*. Values are mean ± SEM. *p<0.05, **p<0.01, ***p<0.001, ****p<0.0001.

**Supplemental Table 1A:** Cytokine values (pg/ml) in cervicovaginal cells co-cultured with live *L. crispatus* (LC), *G. vaginalis* (GV) or non-treated control (NTC)

|  | Ectocervical Cells | | | | Endocervical Cells | | | | Vaginal Cells | | | |
| --- | --- | --- | --- | --- | --- | --- | --- | --- | --- | --- | --- | --- |
|  | NTC | LC | NTC | GV | NTC | LC | NTC | GV | NTC | LC | NTC | GV |
| EGF | ND | ND | ND | ND | ND | ND | ND | ND | ND | ND | ND | ND |
| Eotaxin | 3.1  *(0.80)* | 1.6  *(1.12)* | 4.6  *(0.99)* | 8.5  *(0.90)* | ND | 4.2  *(0.72)* | 6.5  *(0.49)* | 14.4  *(0.74)* | ND | ND | ND | 6.1  *(1.01)* |
| FGF-2 | ND | ND | ND | ND | ND | ND | ND | ND | ND | ND | ND | ND |
| FLT-3L | ND | ND | ND | ND | ND | ND | ND | ND | ND | ND | ND | ND |
| Fractaline | ND | ND | ND | 37.4  *(12.10)* | ND | 6.4  *(3.68)* | 2.8  *(1.64)* | 43.7  *(10.97)* | ND | ND | ND | 17.3  *(3.60)* |
| G-CSF | ND | ND | 29.34  *(9.99)* | 80.72  *(10.00)* | ND | ND | ND | 664.9  *(34.24)* | ND | ND | ND | 95.7  *(9.87)* |
| GM-CSF | 19.7  *(0.13)* | 20.3  *(0.15)* | 71.7  *(2.44)* | 494.1  *(20.62)* | 11.5  *(0.88)* | 18.4  *(0.23)* | 24.7  *(1.27)* | 330.5  *(16.04)* | 14.6  *(0.58)* | 20.4  *(0.16)* | 15.6  *(0.56)* | 353.8  *(32.05)* |
| GRO | 298.5  *(4.89)* | 224.7  *(3.18)* | 774.2  *(55.90)* | 1555  (*134.0)* | 358.7  *(78.2)* | 312.3  *(28.85)* | 1126  *(176.7)* | 15197  *(2048)* | 87.4  *(8.59)* | 53.5  *(12.04)* | 135.6  *(2.51)* | 1645  *(204.6)* |
| IFNa2 | ND | ND | ND | ND | ND | ND | ND | ND | ND | ND | ND | ND |
| IFNgamma | ND | ND | ND | ND | ND | ND | ND | ND | ND | ND | ND | ND |
| IL-10 | ND | ND | ND | ND | ND | ND | ND | ND | ND | ND | ND | ND |
| IL-12p40 | ND | ND | ND | ND | ND | ND | ND | ND | ND | ND | ND | ND |
| IL-12p70 | ND | ND | ND | ND | ND | ND | ND | ND | ND | ND | ND | ND |
| IL-13 | ND | ND | ND | ND | ND | ND | ND | ND | ND | ND | ND | ND |
| IL-15 | ND | ND | ND | ND | ND | ND | ND | ND | ND | ND | ND | ND |
| IL-17 | ND | ND | ND | ND | ND | ND | ND | ND | ND | ND | ND | ND |
| IL-1Ra | 15.6  *(0.46)* | 16.8  *(0.37)* | 122.7  *(8.21)* | 598.0  *(29.75)* | 10.9  *(1.58)* | 12.0  (0.95) | 30.5  *(1.43)* | 227.3  *(11.73)* | 49.3  *(5.20)* | 45.3  *(5.52)* | 98.4  *(4.66)* | 650.8  *(31.70)* |
| IL-1a | 24.7  *(2.03)* | 26.7  *(0.81)* | 333.7  *(35.92)* | 807.6  *(61.98)* | 42.1  *(5.39)* | 43.1  *(3.14)* | 232.8  *(20.49)* | 895.4  *(69.14)* | 40.2  *(3.23)* | 34.2  *(1.92)* | 93.7  *(3.36)* | 423.9  *(20.85)* |
| IL-1b | 0.30  *(0.10)* | 0.50  *(0.14)* | 3.0  *(0.34)* | 14.1  *(1.21)* | 0.87  *(0)* | 0.88  *(0.01)* | 1.8  *(0.11)* | 20.2  *(2.77)* | 0.19  *(0.08)* | 0.50  *(0.22)* | 0.84  *(0.03)* | 8.9  *(0.73)* |
| IL-2 | ND | ND | ND | ND | ND | ND | ND | ND | ND | ND | ND | ND |
| IL-3 | 0.57  *(0.03)* | 0.64  *(0.06)* | 0.50  *(0.03)* | 0.56  *(0.05)* | ND | ND | ND | ND | ND | 0.48  *(0.02)* | 0.30  *(0.03)* | 0.44  *(0.03)* |
| IL-4 | 3.2  *(1.91)* | 1.9  *(0.93)* | 15.1  *(5.89)* | 24.6  *(1.60)* | 5.6  *(1.14)* | 5.5  *(4.22)* | 23.9  *(5.20)* | 79.9  *(7.59)* | 3.4  *(0.87)* | ND | 0.13  *(0.10)* | 26.9  *(3.38)* |
| IL-5 | 0.47  *(0.02)* | 0.51  *(0.05)* | 0.45  *(0.06)* | 0.47  *(0.02)* | ND | ND | ND | ND | ND | 0.02  *(0.02)* | ND | 0.03  *(0.01)* |
| IL-6 | 45.7  *(0.86)* | 68.5  *(2.11)* | 94.9  *(8.28)* | 227.1  *(12.68)* | 12.1  *(1.10)* | 17.4  *(0.42)* | 38.3  *(8.44)* | 260.1  *(4.76)* | ND | 0.20  *(0.03)* | 0.79  *(0.19)* | 23.0  *(1.45)* |
| IL-7 | ND | ND | ND | ND | ND | ND | ND | ND | ND | ND | ND | ND |
| IL-8 | 196.7  *(6.38)* | 169.5  *(0.59)* | 422.0  *(62.68)* | 876.2  *(81.20)* | 138.5  *(3.27)* | 155.2  *(3.57)* | 401.9  *(38.23)* | 2408  *(203.0)* | 37.5  *(2.33)* | 35.4  *(1.72)* | 71.0  *(1.72)* | 875.7  *(63.20)* |
| IL-9 | 0.20  *(0.16)* | 0.19  *(0.12)* | 0.06  *(0.06)* | 0.13  *(0.12)* | ND | ND | ND | ND | ND | ND | ND | ND |
| IP-10 | 17.7  *(0.91)* | 14.4  *(1.39)* | 53.5  *(6.51)* | 75.2  *(5.40)* | 18.9  *(2.12)* | 24.0  *(2.28)* | 462.9  *(131.7)* | 1527  *(163.7)* | 9.3  *(3.03)* | 6.9  *(1.85)* | 45.2  *(7.48)* | 121.0  *(33.68)* |
| MCP-1 | 1.1  *(0.05)* | 1.2  *(0.09)* | 1.3  *(0.16)* | 1.5  *(0.21)* | 1.2  *(0.20)* | 0.71  *(0.14)* | ND | 1.7  *(0.72)* | ND | ND | ND | 1.3  *(0.12)* |
| MCP-3 | 3.6  *(1.14)* | 4.4  *(0.81)* | 3.0  *(0.64)* | 4.6  *(0.04)* | ND | ND | ND | ND | ND | ND | ND | ND |
| MIP-1a | 1.4  *(0.21)* | 1.2  *(0.14)* | 1.6  *(0.06)* | 2.6  *(0.32)* | 0.40  *(0.38)* | 0.66  *(0.25)* | 0.74  *(0.13)* | 2.8  *(0.12)* | ND | 1.2  *(0.30)* | 0.87  *(1.04)* | 4.2  *(0.27)* |
| MDC | ND | ND | ND | 3.7  *(0.98)* | ND | 4.0  *(2.17)* | ND | 5.2  *(1.68)* | 2.2  *(1.63)* | 4.3  *(1.09)* | 2.8  *(0.39)* | ND |
| MIP-1b | ND | ND | ND | ND | ND | ND | ND | ND | ND | ND | ND | ND |
| PDGF-AA | 7.4  *(0.43)* | 6.7  *(0.04)* | 7.6  *(0.53)* | 5.4  *(0.24)* | 29.5  *(4.18)* | 29.2  *(1.72)* | 23.1  *(0.76)* | 15.8  *(0.49)* | 32.2  *(2.38)* | 24.4  *(2.22)* | 26.3  *(0.47)* | 15.7  *(1.75)* |
| PDGF-AB/BB | ND | ND | ND | 7.2  *(3.46)* | ND | ND | 1.2  *(1.51)* | 14.4  *(1.34)* | ND | ND | ND | 11.2  *(2.15)* |
| RANTES | ND | ND | 2.7  *(0.71)* | 3.4  *(0.21)* | 3.1  *(1.22)* | 3.0  *(0.44)* | 21.7  *(3.47)* | 23.5  *(1.77)* | ND | ND | 1.1  *(0.45)* | 3.3  *(0.34)* |
| TGFa | 48.9  *(0.98)* | 47.9  *(0.37)* | 86.6  *(3.29)* | 110.6  *(8.66)* | 40.7  *(1.80)* | 40.9  *(2.32)* | 41.7  *(1.26)* | 68.5  *(2.05)* | 65.6  *(2.23)* | 59.0  *(2.95)* | 72.0  *(4.03)* | 133.5  *(4.73)* |
| TNFa | 0.59  *(0.10)* | 0.71  *(0.20)* | 1.4  *(0.17)* | 26.6  *(3.53)* | 0.39  *(0.09)* | ND | ND | 1.1  *(0.11)* | ND | ND | ND | 16.1  *(1.18)* |
| TNFb | ND | ND | ND | ND | ND | ND | ND | ND | ND | ND | ND | ND |
| VEGF | 41.3  *(0.96)* | 40.3  *(1.09)* | 55.9  *(5.11)* | 41.8  *(3.30)* | 45.5  *(2.29)* | 45.7  *(1.18)* | 48.1  *(3.05)* | 73.6  *(2.32)* | 33.5  *(1.48)* | 23.1  *(3.03)* | 29.0  *(1.35)* | 45.3  *(5.89)* |
| sCD40L | ND | ND | ND | ND | ND | ND | ND | ND | ND | ND | ND | ND |
| TGF-B1 | 9.5  *(1.20)* | 8.5  *(2.61)* | 23.1  *(1.62)* | 25.2  *(0.29)* | 16.0  *(5.15)* | 20.1  *(1.40)* | 42.1  *(1.14)* | 40.0  *(1.13)* | 27.5  *(1.25)* | 21.8  *(3.30)* | 28.9  *(0.66)* | 31.5  *(1.31)* |
| TGF-B2 | 21.7  *(5.06)* | 15.9  *(2.74)* | 19.1  *(1.17)* | 24.4  *(5.24)* | 14.1  *(3.37)* | 13.3  *(0.31)* | 21.5  *(1.50)* | 20.4  *(0.23)* | 16.7  *(0.32)* | 13.7  *(1.61)* | 17.1  *(8.17)* | 19.3  *(0.31)* |
| TGF-B3 | ND | ND | ND | ND | ND | 2.9  *(0.11)* | 3.6  *(0.19)* | 3.2  *(0.06)* | 3.2  *(0.20)* | 0.79  *(0.54)* | 0.54  *(0.24)* | 0.79  *(0.54)* |

*Standard deviation in italics parenthesis below pg/ml

**Supplemental Table 1B:** Cytokine values (pg/ml) in cervicovaginal cells exposed to *L. crispatus* (LC), *G. vaginalis* (GV) bacteria-free supernatant or NYC media alone (NYC)

|  | Ectocervical Cells | | | Endocervical Cells | | | Vaginal Cells | | |
| --- | --- | --- | --- | --- | --- | --- | --- | --- | --- |
|  | NYC | LC | GV | NYC | LC | GV | NYC | LC | GV |
| EGF | ND | ND | ND | ND | ND | ND | ND | ND | ND |
| Eotaxin | 8.6  *(0.70)* | 6.2  *(0.61)* | 10.2  *(0.55)* | 13.1  *(0.94)* | 9.1  *(0.09)* | 15.0  *(0.21)* | 3.6  *(0.24)* | ND | 7.0  *(0.47)* |
| FGF-2 | ND | ND | ND | ND | ND | ND | ND | ND | ND |
| FLT-3L | ND | ND | ND | ND | ND | ND | ND | ND | ND |
| Fractaline | ND | ND | ND | 20.1  *(14.46)* | 10.7  *(1.88)* | 14.9  *(7.42)* | ND | ND | ND |
| G-CSF | ND | ND | 43.3  *(2.99)* | ND | ND | 47.3  *(5.63)* | 60.0  *(1.95)* | ND | 25.3  *(7.89)* |
| GM-CSF | 66.9  *(3.58)* | 84.8  *(6.20)* | 153.6  *(6.43)* | 71.2  *(0.71)* | 33.1  *(0.98)* | 138.6  *(4.39)* | 47.4  *(1.67)* | 106.8  *(4.23)* | 76.1  *(3.25)* |
| GRO | 1304  *(29.08)* | 729.2  *(14.09)* | 1494  *(48.98)* | 4036  *(181.9)* | 2079  *(147.3)* | 8265  *(669.6)* | 1788  *(114.6)* | 715.7  *(55.17)* | 1730  *(75.46)* |
| IFNa2 | ND | ND | ND | ND | ND | ND | ND | ND | ND |
| IFNgamma | ND | ND | ND | ND | ND | ND | ND | ND | ND |
| IL-10 | ND | ND | ND | ND | ND | ND | ND | ND | ND |
| IL-12p40 | ND | ND | ND | ND | ND | ND | ND | ND | ND |
| IL-12p70 | ND | ND | ND | ND | ND | ND | ND | ND | ND |
| IL-13 | ND | ND | ND | ND | ND | ND | ND | ND | ND |
| IL-15 | ND | ND | ND | ND | ND | ND | ND | ND | ND |
| IL-17 | ND | ND | ND | ND | ND | ND | ND | ND | ND |
| IL-1Ra | 13.8  *(0.67)* | 9.2  *(1.21)* | 203.8  *(13.38)* | 12.4  *(1.23)* | 7.3  *(0.49)* | 37.5  *(1.88)* | 44.3  *(1.69)* | 29.7  *(1.58)* | 119.9  *(0.56)* |
| IL-1a | 30.5  *(1.95)* | 31.7  *(2.72)* | 328.9  *(9.45)* | 65.2  *(1.85)* | 46.3  *(1.84)* | 202.0  *(8.53)* | 46.7  *(4.24)* | 48.8  *(3.67)* | 95.4  *(4.12)* |
| IL-1b | 5.8  *(0.26)* | 4.0  *(0.34)* | 8.6  *(0.09)* | 6.2  *(0.15)* | 4.2  *(0.39)* | 7.5  *(0.44)* | 4.1  *(0.22)* | 2.7  *(0.11)* | 6.5  *(0.49)* |
| IL-2 | ND | ND | ND | ND | ND | ND | ND | ND | ND |
| IL-3 | 0.40  *(0.02)* | 0.40  *(0.02)* | 0.41  *(0.01)* | ND | ND | ND | 0.26  *(0.01)* | 0.30  *(0.01)* | 0.32  *(0.02)* |
| IL-4 | 17.0  *(0.56)* | 8.3  *(1.41)* | 17.5  *(0.37)* | 41.2  *(1.73)* | 30.2  *(1.58)* | 64.3  *(1.55)* | 25.7  *(2.91)* | 8.7  *(3.45)* | 23.0  *(1.39)* |
| IL-5 | 0.49  *(0.05)* | 0.47  *(0.06)* | 0.54  *(0.03)* | ND | ND | ND | 0.10  *(0)* | 0.04  *(0.04)* | 0.04  *(0.01)* |
| IL-6 | 979.3  *(29.32)* | 501.1  *(23.09)* | 624.3  *(19.34)* | 574.9  *(1.74)* | 251.3  *(8.87)* | 699.5  *(18.42)* | 272.1  *(12.45)* | 61.6  *(10.94)* | 403.9  *(38.16)* |
| IL-7 | ND | ND | ND | ND | ND | ND | ND | ND | ND |
| IL-8 | 1354  *(34.77)* | 809.4  *(31.56)* | 2075  *(45.77)* | 2677  *(149.6)* | 1045  *(40.89)* | 4145  *(69.80)* | 1092  *(38.46)* | 545.2  *(24.14)* | 2877  *(261.5)* |
| IL-9 | ND | ND | ND | ND | ND | ND | ND | ND | ND |
| IP-10 | 45.2  *(0.88)* | 4701  *(4.45)* | 94.3  *(4.32)* | 63.9  *(1.86)* | 53.4  *(1.11)* | 135.2  *(14.38)* | 46.28  *(0.24)* | 40.10  *(2.46)* | 67.41  *(11.29)* |
| MCP-1 | 1.9  *(0.40)* | 1.8  *(0.19)* | 1.7  *(0.19)* | 1.5  *(0.46)* | 0.94  *(0.46)* | 1.4  *(0.47)* | 1.3  *(0.18)* | 1.2  *(0.24)* | 1.5  *(0.11)* |
| MCP-3 | 4.5  *(0.99)* | 4.9  *(1.28)* | 4.4  *(0.50)* | ND | ND | ND | 1.8  *(0.53)* | ND | 4.1  *(2.77)* |
| MIP-1a | 1.4  *(0.31)* | 1.2  *(0.13)* | 3.6  *(0.22)* | 1.8  *(0.08)* | 1.2  *(0.26)* | 2.2  *(0.15)* | 4.7  *(1.25)* | 4.0  *(1.01)* | 5.6  *(0.36)* |
| MDC | ND | ND | ND | 5.3  *(3.72)* | 5.2  *(2.29)* | 4.3  *(2.88)* | ND | 4.5  *(0.25)* | 5.5  *(0.60)* |
| MIP-1b | ND | ND | ND | ND | ND | ND | ND | ND | ND |
| PDGF-AA | 5.2  *(0.26)* | 3.5  *(0.12)* | 6.4  *(0.33)* | 18.4  *(0.39)* | 15.5  *(0.77)* | 22.6  *(1.09)* | 18.5  *(0.86)* | 16.7  *(0.70)* | 17.6  *(0.71)* |
| PDGF-AB/BB | 5.4  *(1.20)* | 6.2  *(1.10)* | 13.6  *(1.82)* | 10.8  *(1.51)* | 8.7  *(1.46)* | 27.9  *(2.86)* | 10.0  *(2.29)* | 11.5  *(1.03)* | 21.1  *(1.33)* |
| RANTES | ND | ND | 4.9  *(0.67)* | 7.5  *(1.02)* | 2.8  *(0.95)* | 16.4  *(1.14)* | ND | ND | 2.7  *(0.50)* |
| TGFa | 99.0  *(1.92)* | 61.5  *(3.86)* | 163.3  *(7.79)* | 132.0  *(5.08)* | 77.2  *(3.84)* | 151.1  *(7.95)* | 228.1  *(17.63)* | 141.2  *(2.84)* | 266.3  *(10.57)* |
| TNFa | 1.0  *(0.09)* | 1.8  *(0.29)* | 4.1  *(0.16)* | 0.23  *(0.08)* | ND | 0.18  *(0.17)* | 1.0  *(0.16)* | 0.44  *(0.13)* | 3.3  *(0.69)* |
| TNFb | ND | ND | ND | ND | ND | ND | ND | ND | ND |
| VEGF | 81.6  *(2.01)* | 81.0  *(9.17)* | 82.2  *(3.95)* | 84.2  *(5.04)* | 180.3  *(6.86)* | 102.5  *(2.65)* | 67.3  *(2.29)* | 223.0  *(22.14)* | 85.3  *(1.93)* |
| sCD40L | ND | ND | ND | ND | ND | ND | ND | ND | ND |
| TGF-B1 | 60.1  *(0.79)* | 65.7  *(3.03)* | 75.5  *(4.98)* | 81.3  *(2.36)* | 34.0  *(1.17)* | 94.1  *(2.10)* | 82.0  *(5.08)* | 85.6  *(5.27)* | 75.0  *(0.87)* |
| TGF-B2 | 59.7  *(0.48)* | 62.3  *(2.67)* | 46.0  *(2.68)* | 48.7  *(0.45)* | 45.7  *(0.48)* | 45.9  *(1.29)* | 41.7  *(1.48)* | 37.9  *(1.08)* | 40.8  *(1.70)* |
| TGF-B3 | ND | ND | ND | 4.1  *(0.21)* | 3.8  *(0.10)* | 3.6  *(0.19)* | 1.3  *(0.29)* | 0.77  *(0.17)* | 1.2  *(0.78)* |

*Standard deviation in italics parenthesis below pg/ml

**Supplemental Table 2A:** Number of altered cytokines after exposure to live bacteria

| Live Bacteria | Ectocervical | Endocervical | Vaginal |
| --- | --- | --- | --- |
| L. crispatus |  |  |  |
| Total # altered | 2 | 3 | 4 |
| Analytes ↑ | 1 | 1 | 2 |
| Analytes ↓ | 1 | 2 | 2 |
|  | | | |
| G. vaginalis |  |  |  |
| Total # altered | 16 | 19 | 22 |
| Analytes ↑ | 15 | 18 | 20 |
| Analytes ↓ | 1 | 1 | 2 |

**Supplemental Table 2B:** Number of altered cytokines after exposure to bacteria-free supernatants

| Bacteria-free Supernatants | Ectocervical | Endocervical | Vaginal |
| --- | --- | --- | --- |
| L. crispatus |  |  |  |
| Total # altered | 10 | 16 | 11 |
| Analytes ↑ | 2 | 4 | 4 |
| Analytes ↓ | 8 | 12 | 7 |
|  | | | |
| G. vaginalis |  |  |  |
| Total # altered | 18 | 18 | 13 |
| Analytes ↑ | 17 | 18 | 12 |
| Analytes ↓ | 1 | 0 | 1 |

**Supplemental Table 3:** Number of cytokines altered after blocking TLR2

| Live Bacteria | Ectocervical | Endocervical | Vaginal |
| --- | --- | --- | --- |
| LC vs LC + anti-TLR2 AB |  |  |  |
| Total # altered | 9 | 0 | 1 |
| Analytes ↑ | 6 | 0 | 1 |
| Analytes ↓ | 3 | 0 | 0 |
|  | | | |
| GV vs GV + anti-TLR2 AB |  |  |  |
| Total # altered | 8 | 11 | 4 |
| Analytes ↑ | 0 | 2 | 2 |
| Analytes ↓ | 8 | 9 | 2 |
